# There is ‘no cure for caregiving’: the experience of women caring for husbands living with Parkinson’s disease

**DOI:** 10.1080/17482631.2024.2341989

**Published:** 2024-04-24

**Authors:** Dawn R. White, Patrick A. Palmieri

**Affiliations:** a College of Graduate Health Studies, A. T. Still University, Kirksville, MO, USA; b Benerd College, University of the Pacific, Stockton, CA, USA; c South American Center for Qualitative Research, Universidad Norbert Wiener, Lima, Peru; d College of Nursing and Health Sciences, Excelsior University, Albany, NY, USA; e EBHC South America: A JBI Affiliated Group, Escuela Posgrado, Universidad Nortbert Wiener, Lima, Peru; f Center for Global Nursing, Texas Woman’s University, Houston, TX, USA

**Keywords:** Caregivers, caregiver burden, emotional exhaustion, Parkinson’s disease, phenomenology, qualitative research

## Abstract

**Background:**

Parkinson’s disease is a progressive neurodegenerative disorder. The majority of the nearly 9 million people living with Parkinson’s disease are men. As such, caregiving is often assumed by wives as the disease progresses. However, there is little research about the lived experience of wives as they transition to caregivers.

**Objective:**

To describe the lived experience of wife caregivers of male spouses living at home with Parkinson’s disease.

**Methods:**

A descriptive phenomenological study. Semi-structured interviews were recorded and transcribed for analysis in Atlas.ti using Colaizzi’s method.

**Results:**

Thirteen women, aged 50 to 83 years, were interviewed. Five themes emerged from the analysis, (1) caregiver who? (2) taking it day by day, (3) not sure what to do next, (4) just too much, and (5) caring is your soul’s growth, to support the central theme “there is no cure for caregiving.”

**Conclusion:**

Transitioning from wife to caregiver was a gradual but difficult process. Although the wife caregivers wanted to be part of the health care team, they remained outsiders. Clinicians need to recognize the wives as care coordinators linking medical management with home care. Policy makers need to develop reimbursement models that provide wife caregivers with support groups, education programs, and telemental health services.

## Introduction

Parkinson’s disease is a neurological disorder that alters motor and non-motor functions of people. The disease was discovered in 1817 by Dr. James Parkinson, and 22 people were diagnosed by 1855 (Dorsey, Elbaz, et al., 2018; McDonald et al., 2018). Notable symptoms, later referred to as “shaking palsy or paralysis agitans” (McDonald et al., 2018), included tremors, bradykinesia, rigidity, and progressed to cognitive deficits such as executive dysfunction (Cerri et al., 2019; Fang et al., 2020).

Globally, there is an estimated 8.5 million people living with Parkinson disease (James et al., 2018), with a mortality rate 1.5 greater than the general population (Marras et al., 2018). Although the prevalence of Parkinson’s disease increases with age (Marras et al., 2018; Willis et al., 2022), research suggests that genetics, including 20 causative genes (Funayama et al., 2023), and industrial chemical exposure, such as working in environments with pesticides (Dorsey, Sherer, et al., 2018), may be other contributing factors. People living with Parkinson’s disease are twice as likely to be men than women (Baldereschi et al., 2000; Willis et al., 2022), possibly linked to prior occupational chemical exposure (Cao et al., 2019), but women have a higher mortality rate and more rapid disease progression (Cerri et al., 2019). Men living with Parkinson’s disease are less likely to access paid formal caregiver services than women, and women are more likely to serve as caregivers (Dahodwala et al., 2018). This may result from men having greater access to an informal caregiver than women (National Alliance for Caregiving, 2020) and women being more likely to outlive their male spouses (Rochelle et al., 2015). There are other potential advantages influencing informal caregiving from a spouse such as reduced caregiving costs and improved quality of life (Boersma et al., 2017; Prizer et al., 2020).

Most Parkinson´s disease caregivers are female spouses (Cianfrocca et al., 2020; Martinez-Martin et al., 2023; Walga, 2019) who transition from wife to caregiver as the disease progresses. Eventually, the wife only identifies as a caregiver and not a wife (Vatter et al., 2018). Wife caregivers accompany their spouses to medical appointments as they try to bridge the widening gap between the patient and the physician (Biderman et al., 2021). However, the existing literature suggests physicians have limited knowledge about aiding caregivers (Schwartz et al., 2020). Thus, wives experience pressure and fatigue as they become faceless people visiting the physician with their husband (Dekawaty et al., 2019; Kang et al., 2020). While previous studies reported quantitative findings about Parkinson’s disease caregivers, few provided insights about the lived experiences of wife caregivers (White & Palmieri, 2022).

## Background

The symptoms of Parkinson’s disease begin slowly and become more noticeable with time. As the symptoms advance, patients begin to miss work, require additional medical care, and need assistance with activities of daily living (Smith et al., 2019). The Hoehn and Yahr (1967) scale is commonly used by neurologists and movement specialists to rate the severity of Parkinson’s disease. The scale progresses from stage 1 to stage 5 as the disease advances in severity. The disease stage correlates with the amount of caregiving required for patients to engage in activities of daily living (Skorvanek et al., 2017).

### Caregiver role

Caregivers often think of others first and continue forward in caring for other people at all costs (Prado et al., 2020). To make the patient’s life easier, a caregiver typically assists with all aspects of living, such as activities of daily living (Lennaerts-Kats et al., 2020). However, these duties can decrease the quality of life of the caregiver (Vatter et al., 2018), increasing stress (Torny et al., 2018), physical pain (Biderman et al., 2021), and financial hardship associated with medical expenses (Boersma et al., 2017). Their responsibilities create a multitude of feelings that can lead to “frustration, resentment, anger, sadness and a worry for the future” (Vatter et al., 2018, p. 604). Historically, caregivers have not engaged in self-care activities which is more specific for wives as caregivers. Anticipating the difficulties associated with the progressive terminal condition, the caregivers begin to lose sleep, experience the onset of grief, and cope with depression (Turney & Kushner, 2018).

Caregiving places a wife in a role that requires them to exert extreme effort for the physical and mental care of another, while feeling exhausted (Thieken & van Munster, 2021). Women caregivers are usually aged 65 years or older, and have physical limitations associated with ageing (Biderman et al., 2021). As their role shifts into being a caregiver, feelings of disconnect become common. According to Smith and Shaw (2017) women reported feeling “disharmony, disequilibrium, dis-ability, and dis-ease which incorporates a loss of the familiar world” (p. 14), to the extent of feeling displaced from their life. To break the monotony of work and stress, caregivers should pursue outside activities that increase their sense of meaning and help their spouse with socialization (Prado et al., 2020). When successful, outside socialization can offset feelings of stress and reset the psyche of both parties (Perepezko et al., 2019). Unfortunately, attendance at functions or group activities typically becomes more about their spouse moving and socializing and less about the wife finding an outlet for their needs. Wife caregivers sacrifice their time and social identity at functions geared towards their husband (Prado et al., 2020). Having conflicting emotions can increase caregiver burden but, conversely, also increase the benefits they perceive resulting from their caregiving (Geerlings et al., 2023).

### Caregiver burden and benefit

Caregiver burden is a common among those providing care for Parkinson’s disease patients (Juneja et al., 2020). The slow disease progression results in caregiver uncertainty about their husband’s life expectancy (Geerlings et al., 2023). Often, the husband living with Parkinson’s disease is unaware of the burden their care places on their wife as a caregiver (Balash et al., 2019). Burden results from caregiver discomfort due to “emotional, financial, social, physical, and spiritual functions” when providing direct care (Smith et al., 2019). An example of a burden is the internal dilemma of caregivers when deciding whether they should care for their spouse or themselves. Typically, they choose their spouse’s needs and set theirs aside for a later time that never comes. This cycle is considered the caregiver career and can result in overwhelming exhaustion and burnout (Savundranayagam & Montgomery, 2010). Exhaustion, which is typically associated with burden, occurs when caregiving becomes a full-time job (Theed et al., 2017). Symptoms of exhaustion include tense muscles, emotional drain, and stress that last longer than 6 months, which leads to lower quality of life for the caregiver (Kang et al., 2020). Similarly, caregivers can experience depersonalization which results in severe depression (Kist Bakof et al., 2021).

Research often focuses on caregiver burden (Klietz et al., 2021) and rarely on the benefits (Perry et al., 2022). Because of their willingness to provide extended and loving care, caregivers experience personal growth and have a sense of purpose. They also develop compassion while learning patience and understanding (Walga, 2019). If done correctly, there will be a mutual respect with a stronger relationship between caregiver and patient (Champagne & Muise, 2022; Geerlings et al., 2023). Although historical research on burden has mostly been quantitative, caregivers should have the opportunity to convey their lived experiences in their own words and express how caregiving has affected their lives (Klietz et al., 2021). Having such a platform would allow them to build support from their families and communities.

### Purpose of the study

The literature frequently refers to caregivers who are not educated in the health sciences and uncompensated for their care as informal caregivers (Hand et al., 2019; Navarta‐Sánchez et al., 2020). This term can be stigmatizing for women caring for a family member and engaging in work similar to staff at health care institutions, such as nursing assistants and care partners. Therefore, the current study defined a Parkinson disease caregiver as any person caring for another person with Parkinson disease that limited their ability to engage in activities of daily living. The purpose of the study was to describe the lived experience of women caregivers of male spouses living at home with Parkinson’s disease.

## Methods

### Study design

The current study used a descriptive phenomenological design (Moustakas, 1994). Descriptive phenomenology is a qualitative method that explains “information and insight do not come from large amounts of data, but emerges from an intense study of experiences” (Husserl, 2012). The study design permits the contextualization of the individual lived experience of a wife caregiver for a husband living at home with Parkinson´s disease as a shared phenomenon (Starks & Brown Trinidad, 2007) to uncover the underlying social realities useful for generating knowledge to improve health care (Lopez & Willis, 2004). This approach provided the wives an opportunity to share their lived caregiving experiences through conversational interviews. The interviews resulted in rich data that described the spirit of their spoken word based on previous life experiences (Rodriguez & Smith, 2018). The approach focused on asking the women, “How did we get here? What did we learn?” (Neubauer et al., 2019) and “What was it like?” (van Manen, 2017).

### Setting and participants

Participants were recruited from the membership of the Colorado Parkinson Foundation. Using a purposive sampling technique (Gentles et al., 2015), variation was sought in the sociodemographic profile of the participants. Participants meeting the inclusion criteria were asked to provide sociodemographic information and their informed consent. Criteria for inclusion were women caregivers of male husbands diagnosed with Parkinson’s disease stages 2 to 4 as assessed with Hoehn-Yahr staging (Hoehn & Yahr, 1967), age 50 years or older, living at home in Colorado (cohabitating), and speak and understand English. Stage 1 was excluded as patients are often unaware of their diagnosis or they rarely require caregiving and stage 5 was excluded as patients are often bed-ridden and receive formal nursing care (Iwasa et al., 2021). Participants were recruited until data saturation was achieved (Sandelowski, 1995); the point when no new information was found in the data (Saunders et al., 2018). The number of participants anticipated to achieve saturation for this study was 8 to 16 (Guest et al., 2020; Mason, 2010).

### Data collection

A semi-structured interview guide was created during the literature review and focused on questions related to caregiving. The 14 interview questions (Table I) were slightly modified during data collection as additional areas for inquiry were identified (Roberts, 2020). Interviews were completed in a conversational style (Alase, 2017), which allowed the participants to feel comfortable during their interview. This interview style provided flexibility for participants to explore their experiences and realize the underlying reasons describing the phenomenon of being a caregiver to their spouse (Barriball & While, 1994). The interviews were conducted through Zoom©, audio recorded, and sent to an outside company for transcription. The Zoom© platform provides digital videoconferencing appropriate for qualitative research when face-to-face interviews are not possible (Archibald et al., 2019; Gray et al., 2020; Krouwel et al., 2019; Lobe, 2017; Lobe et al., 2020; Oliffe et al., 2021). During the interview, field notes about participant statements (Phillippi & Lauderdale, 2018) and pertinent words were documented for analysis. At the beginning of the interview, demographic information was collected and included age, children, education, work status, medical training, years of caregiving, years of marriage, work situation, duration of disease, relative location, household income, and a self-assessment of caregiving hours per week.

**Table I.** Semi-structured interview guide with evidence sources.

Interview Question	Evidence Sources	Construct(s)
Tell me about your experiences of caring for your husband with Parkinson’s disease.	Open the conversation with the participant	Grand question
Please describe your experiences working with your husband’s physicians. How did they help or hinder? (Prompt: How are you addressed during visits? How can they help?)	Boersma et al. (2017) Dekawaty et al. (2019)	Wife caregiver Caregiver burden Caregiver benefit
What was your experience when your husband was first diagnosed with Parkinson’s disease? (Prompt: How did you feel? What did you think?)	Hoogland et al. (2019) Schwartz et al. (2020)	Wife caregiver
What disease symptoms are most challenging for you to manage? How do you deal with them? (Prompt: What might make these easier for you to manage?)	Boersma et al. (2017) Smith et al. (2019)	Caregiver burden Fatigue
Where do you find your strength? Describe your inner strength. (Prompt: What helps you continue caring?)	Boersma et al. (2017)	Caregiving Wife caregiver
Please describe what you normally do during the time you are caregiving. (Prompt: What does caring mean in the context of your work with your husband?)	Kist Bakof et al. (2021) Kang et al. (2020) Theed et al. (2017)	Caregiving Fatigue Wife caregiver
What is it like to go from being a wife and spouse to a full-time caregiver for a person living with Parkinson’s disease?	Balash et al. (2019)	Caregiving Caregiver burden
Please describe your experience related to the progression of your husband’s disease. (Prompt: What has been difficult?)	Juneja et al. (2020) Smith et al. (2019)	Caregiving Wife caregiver
As a caregiver for a person living with Parkinson’s disease, how your life has changed? (Prompt: Good, not so good, bad?)	Smith et al. (2019)	Caregiver burden Caregiver benefit
What is your experience with outside support you have received from the community, agencies, friends, and/or health providers?	Dekawaty et al. (2019) Turney et al. (2018) Walga (2019)	Caregiving Caregiver burden
What are your experiences with adapting your life to your spouse’s Parkinson’s disease?	Dekawaty et al. (2019) Hellqvist et al. (2020)	Caregiving
In your own words, explain how you deal with the burden, or stress, of caring for your husband. (Prompt: Are there any benefits?)	Walga (2019)	Caregiver burden Caregiver benefit
Please explain in your own words what drives you to continue caring for your spouse. (Prompt: What are your motivators?)	Champagne et al. (2022)	Caregiver benefit Wife caregiving
Is there anything else you would like to add?	Closing remarks	Final question

### Data analysis

Immediately after transcription, the interview documents were uploaded into the data analysis package Atlas.ti (ATLAS.ti Scientific Software Development GmbH). The transcripts were read multiple times and compared with the audio recordings to gain familiarity with the content and check for accuracy. The transcripts were then analysed using Colaizzi’s (1973, 1978) seven-step process (Pardell-Dominguez et al., 2021), which is a comprehensive method for analysis of descriptive phenomenology data (Morrow et al., 2015). The steps of this process include the following: (1) gaining familiarization with the data (2), identifying significant statements (3), formulating meanings (4), clustering themes (5), developing an exhaustive description (6), producing the fundamental structure, and (7) seeking verification of the fundamental structure (Pardell-Dominguez et al., 2021; White & Palmieri, 2022). The analysis resulted in a description of the complex phenomenon called caregiving across multiple lived experiences (Sundler et al., 2019). Once all transcribed interviews were coded, the data were clustered into themes and subthemes relevant to caregiving.

### Trustworthiness

Trustworthiness refers to the credibility, transferability, dependability, and confirmability (Guba & Lincoln, 1994; Lincoln & Guba, 1985) of a study such that the findings “support the argument that the inquiry’s findings are worth paying attention to” (Elo et al., 2014) in an accurate and concise manner (Rose & Johnson, 2020). To address credibility, member checking was performed by returning the analysis to participants for validation. Participants reviewed the document and provided feedback with some clarifying but not contradictory comments. Dependability was established through the published protocol (White & Palmieri, 2022), which added to the formalization of the audit trail for decisions (Sanders, 2003). Confirmability was achieved through bracketing to limit researcher bias (McGinley et al., 2021) and having two investigators analyse the transcripts. Each researcher conducted an independent data analysis, and through iterative discussion, consensus was achieved. The study procedures incorporated additional strategies for maximizing rigour and limiting bias (Phillippi & Lauderdale, 2018), including journaling, bracketing, coding checks, team meetings, member checking, and peer debriefing with outside researchers during data collection and analysis. An expanded data file with additional quotes provides a thick description for the subthemes and themes. Finally, the study is reported in accordance with Standards for Reporting Qualitative Research guidelines (O’Brien et al., 2014), and the protocol was previously reported (White & Palmieri, 2022).

## Results

Thirteen women completed interviews, which lasted 30–60 minutes and averaged 49 minutes. All the women were married and caring at home for husbands diagnosed with Parkinson’s disease. They lived in Colorado Springs, Colorado, and were aged 50–83 years. Participant demographic data are presented in Table II.

**Table II.** Demographic characteristics of women caregivers of male spouses living with Parkinson’s disease.

Pseudonym	Age	Education	Years Married	Job Status	Children	Relative <20 Miles	Caregiving Years	Caregiving Hours (weekly)	Medical Training
Carnation	80–89	Bachelor	51	Not Working	1	Yes	12	80 +	No
Crocus	50–59	Bachelor	55	Not Working	2	Yes	3	80 +	No
Daisy	70–79	Bachelor	14	Not Working	2	Yes	9	50–59	No
Iris	70–79	Associate	52	Not Working	2	No	2	50–59	No
Lavender	60–69	Bachelor	8	Not Working	2	Yes	11	50–59	No
Lilac	60–69	Master	30	Not Working	1	No	8	50–59	No
Lily	60–69	Bachelor	25	Not Working	0	Yes	18	80+	No
Magnolia	70–79	Master	54	Not Working	1	Yes	5	80+	No
Pansy	80–89	Bachelor	60	Not Working	2	Yes	1	50–60	No
Petunia	70–79	Bachelor	57	Not Working	2	Yes	17	80+	No
Poppy	60–69	Associate	34	Not Working	3	Yes	12	80+	No
Rose	70–79	Bachelor	52	Not Working	3	Yes	4	80+	No
Violet	70–79	Bachelor	44	Not Working	2	No	20	80+	No

Analysis of interviews resulted in 113 codes that were consolidated into 15 subthemes. The subthemes were then organized into 5 themes (Table III) with a central theme of “There is no cure for caregiving.” The 5 themes included the following: (1) caregiver who? (2) taking it day by day (3), not sure what to do next (4), just too much, and (5) caring is your soul’s growth. The 15 subthemes offered insight into the participants’ lived experiences and included early years; more caring less curing; living in the shadows; golden years; who’s gonna help me; climbing the mountain; it’s my way now; nursing without a degree; emotional rollercoaster; physical and mental overload; I’m just so angry; managing the craziness; please, God, help me; finding my inner strength; and how can I help you. An expanded data file that supports the themes and subthemes is provided in Supplemental File 1.

**Table III.** Themes and subthemes from phenomenological analysis of interview data.

Themes	Subthemes
Caregiver who?	Early years More caring less curing Living in the shadows
Taking it day by day	Golden years Who’s gonna help me? Climbing the mountain
Not sure what to do next	It’s my way now Nursing without a degree Emotional rollercoaster
Just too much	Physical and mental overload I’m just so angry Managing the craziness
Caring is your soul’s growth	Please, God, help me Finding my inner strength How can I help you?

### There is no cure for caregiving

Participants saw caregiving as a responsibility that required emotionally, psychologically, and physically demanding work to support their husbands as the Parkinson’s disease advanced. The work included feeding, dressing, transporting, and managing physician appointments. As a result, caregivers felt there needed to be a cure for caregivers. One woman stated:

I don’t know if there’s anything in any study that could help the caregiver. What could you people possibly do for a caregiver? I don’t know. I have my son-in-law coming in and relieving me sometimes, which is the only thing that you need—a break. I thank God for that. I do have a break, but there is no cure for caregiver. There is no cure.

(Rose)

The cure for many problems associated with caregiving for a husband living with Parkinson’s disease was shared by the wives as the collective experiences organized in the next sections.

### Theme 1: caregiver who?

The caregiver who? theme described how caregivers are lost in the advancing disease process. Most indicated that shifting neurologists to look for the right doctor did not work. One woman, Iris, stated, “We changed to a different neurologist, and we like this one really well, but I don’t think either of them have really addressed—I don’t know. The caregiver.”

### Early years

Having their spouse diagnosed with a neurodegenerative disease with no known cure was as frightening for the participants as for their husbands. Despite the slow disease progression, the Parkinson’s disease was described by the participants as becoming a third person in their relationship. Many were unprepared for a diagnosis that they had difficulty understanding. Lilac stated, “I was angry at that doctor. I thought, you’ve taken a vow to help people. Like, couldn’t you have been kinder or more encouraging?” She later added, “I kind of denied and didn’t believe the doctor. And the doctor was saying, “No, you can’t drive anymore, buddy. There’s nothing we can do to help you; it’s all downhill from here.”

After the initial feelings of anger, participants transitioned to denial about the disease followed by fear when they realized what was happening. Rose stated, “I was in denial until I couldn’t deny it anymore.” Similarly, Poppy said, “Fear. I think fear was the biggest. What was coming? How would I handle it? Where was my support going to come from? Would I have time for my own self? How was his life going to change? How would he handle that as well? Because neither of us knew anybody with it.”

Throughout their experiences with their husband’s diagnosis, they often commented about the physicians providing no encouragement for the future, which caused them to feel distraught. Petunia said, “He [the doctor] just told him everything, everything that’s going to shut down, didn’t give him any encouragement as to how he would be able to go ahead and live a fairly normal life. So, my husband was quite distraught.” Despite these feelings, participants tried to make sense of the diagnosis by figuring out how it would change their lives. They were primarily concerned about what needed to be done. As Lilac stated:

I think it’s a safety mechanism in my own head, to kind of think, well, can I cope with everything alone when he leaves me? And what do I do if he gets to that fifth stage where he’s on a feeding tube, and he can’t speak and he can’t get himself in and out of bed? And where’s the adage where I can’t any longer cope with it by myself?

To better understand how the medical diagnosis would impact their lives, some participants began doing research. Petunia said, “I thought, okay, here we go. The first thing I did, I’m a type A personality, so as soon as I got home, I started researching everything I could on it, and figuring out what we could, and then trying to figure out finding a different doctor.” Poppy described her search for understanding because of lack of information from the physician at the time of diagnosis:

His neurologist, as we went in and they had already done all the MRIs [magnetic resonance imaging] to see if there were brain tumors and everything as they normally do it, we came in and he [the doctor] looked at him [her husband] and he said, “Look, pal, you’ve got Parkinson’s.” We got no pamphlets. We got no written information. We got no direction as to where to look for information or research.

Poppy added that because her and her husband had a history of being “big researchers” they investigated “what does this disease mean for our life.” In general, this sense-making process was lengthy, and, in many cases, participants pursued it privately to avoid worrying their husbands. As Violet conveyed:

I went to the library and brought home every book I could on Parkinson’s, tucked it in a drawer, and read it when [my husband] wasn’t around. He does not process; he does immediate head-on like I do. I got myself informed and kind of ready. It took me about three years to swallow the fact that he had Parkinson’s and what the future would look like. After that, I kind of gripped it well. That’s how we started.

### More caring less curing

The women described poor experiences with their husbands’ physicians regarding caregivers’ needs. The physicians told the participants there was no cure for Parkinson’s disease. Over time, the participants began to understand this reality. Because there are no medical interventions for Parkinson’s disease, physician appointments focused on physical assessments of their husbands. The participants noted a lack of caring for the entire person and addressing their husband´s other needs related to Parkinson’s disease. Violet stated, “The doctors just pretty much deal with what’s right in front of them. They don’t go beyond there other than to say, ‘You need help, go find it.’” During the medical appointments, the physicians made many assumptions about the situation. This frustration was expressed by Poppy who stated, “The hardest part is getting the doctors to have more time to listen to what’s actually going on and not assume everything is Parkinson’s.”

Participants also expressed concern about being excluded from the process. This sole focus on the patient and lack of acknowledgement of other people affected by the diagnosis was highlighted in statements like the following statement from Lily:

[It’s all] about him, what’s coming, what needs to be done, yeah. No, the caregiver has really never come into this. As far as concentrating his [physician] practice, the caregiver doesn’t really enter in unless that’s an integral part of one of the problems that that person’s having, is because a caregiver is in burnout, then he’ll address it.

To address the lack of acknowledgement participants believed the physicians needed to be more aware of community resources and recommend them to caregivers. Participants believed such information would really help people living with Parkinson’s disease as suggested by Daisy in following statement:

It would be great to compile all the data of what’s available in our community for different illnesses and print up information and get it to the doctors because you’re left on your own to find out. And I know it’s our responsibility, but I think the doctors have a responsibility to help their patients through the journeys of their illnesses. So, I think that would be very beneficial.

### Living in the shadows

Because the work of caregiving demanded more hours than a full-time job, the participants felt they lost track of who they were. As time passed, they began to feel overwhelmed and eventually exhausted. From the physician’s viewpoint, they were responsible for organizing and keeping track of their husband’s progress, but the physician often failed to acknowledge their needs or personal concerns. This disregard for the individual was expressed by Violet who stated, “The health providers give me lists, say, ‘Go find something.’” Some participants reported that conversations between the physician and their husband transpired as if they were not even in the room. As Carnation pointed out, “I wasn’t really impressed with him because when we would go in for an appointment all of his conversation and questions were between him and my husband. And it was like I wasn’t even there.” Other participants indicated that the physician did sometimes include them by having a sidebar conversation with them. The frustration of the occasional inclusion of participants and acknowledgement of their presence during appointments was expressed by Lavender who stated, “As a sidebar issue, maybe once or twice, but not on a regular basis. The only time I get asked that is when I go to the doctor, for me, not necessarily for him.”

Although the participants were mostly ignored by their husband’s physician, they generally accepted that behaviour without protest because they believed their role as the caregivers was not as important as the care of their husbands. Daisy explained this viewpoint as follows:

It’s hard because being the caregiver. That’s your main focus. And you don’t really think about yourself very much. You really don’t because when you’re watching a loved one you push your feelings aside because you want them taken care of and you want to make sure they have everything they need, and you don’t….I don’t really think about it that much, about myself that much. So, it’s hard to talk about it. It’s been kind of frustrating when they don’t listen to you. I don’t think I know more than them, but they just don’t listen.

### Theme 2: taking it day by day

Patients and families living with Parkinson’s disease never know what the next day will bring, so participants frequently indicated that they had to live day by day. Before the Parkinson’s disease diagnosis, they thought about what their retirement years would bring; however, after the diagnosis, their thoughts changed to who will help them and how they would climb the mountain called Parkinson’s disease. This sense of living day by day was expressed by Carnation who said, “There’re days when I’m just exhausted. But I have learned that as I’m getting older, I can’t do as much. I don’t have the energy, and so I have to do what I have to do.”

### Golden years

Because of the Parkinson’s disease diagnosis, participants realized their retirement years would not look like what they imagined. As stated by Carnation, “This was not the way I expected to spend my retirement years because we haven’t been able to travel and go places a lot. Life was just all different then. And you just have no idea that that’s all going to go away and there’s going to be a different lifestyle.” Another woman, Daisy, said, “It’s affected both our lives because we can’t do the things we want to do.” Some participants wanted to try living the best they could during their retirement despite the diagnosis, but sometimes their husbands preferred to stay home. As Lily stated with frustration, “He doesn’t ever want to do anything. If he had his way, he would just stay at home and do nothing. I’m not ready to give up on life yet.” Similarly, other participants reported they were able to enjoy retirement with some limitations. For example, Iris stated, “I just feel like we need to enjoy what freedom we do have because in a few years it could be worse.” Similarly, Magnolia highlighted the nature of caregiving:

I guess we both thought we’d be able to travel a little more in retirement. But because of his limitations, the only travel we’ve done is day trips around Colorado, not long-term trips. Probably, that’s the one thing we thought we would be able to do more of is travel and that hasn’t happened. But again, it is what it is.

### Who’s gonna help me?

Participants indicated that support came from various places and many people. Families, especially those who lived close to the participants, and support groups helped with the physical and emotional care of their husbands. Iris said, “We have two daughters. One’s in Oregon and one’s in Denver. And the one in Denver, her and her husband, they’re very good about anytime we need help. They said just let them know and they’ll come down and help us.” However, even when family lived close, participants indicated that getting help could be difficult because everyone was busy and did not always have time for visits. Carnation stated, “We do have a son here in Colorado Springs, but he’s still across town and works and has kids and he does things to help when I need.”

Some participants reported speaking with family members who had caregiver experience. They described the discussions as working in the same profession. Lavender stressed the importance of this common ground, saying, “I’ve got my brother who’s also a caregiver and he was a medical assistant as well. So, we can talk shop. We can talk. We can relate in almost every way.” Other participants indicated that virtual discussions about their situation with family members were impersonal because it was not possible to share their experiences through Zoom. As stated by Lilac, the online platform made it difficult for others to really grasp their reality “The problem with just doing it on Zoom is they can’t. They say, ‘Oh, you look so good. Everything’s so good. You’re great.’ They don’t, like, live with it. And so, then it’s, like, hard for them to understand.”

Most participants accessed support groups to help them cope with caring for their husbands. Sometimes friends and family helped them locate these groups. Crocus described a nationwide programme she joined on a family member’s recommendation: “They have recommended that I join this caregiver program, which is nationwide and what I have seen, so what I was looking for at the time was some counseling. And we are beginning to get to that where we can do some online counseling.” In some situations, finding the right group for support improved the ability of caregivers to cope with their problems and concerns. As several women were caring for husbands with prior military service, they spoke about accessing the support group at Veterans Affairs (VA) for assistance. For example, Crocus described her experience.

Last fall, we were able to get involved with the VA. The VA has what they call a respite program. They would do the grocery shopping or take him to an appointment if they had the right driver’s license. And they would do some stuff around the house for me, but a lot of it was just some light stuff. And that helps.

All the participants accessed the local Parkinson’s disease support group. Most participants reported important benefits from knowing other people who were experiencing the same things because of Parkinson’s disease. Lily stated, “The support group. Everybody’s in the same boat and camaraderie really helps a lot to know that you’re not alone and everybody’s facing these things.” In addition to the beneficial services and assistance from the support group, many participants also reported making new friends. Rose said, “We made fabulous friends here and they love [my husband]. They come over because Thursday is Canasta Day in my house, and they won’t let me leave him.” Some participants reported they had become incredibly involved in the support group, such as serving on the governance board. Petunia said, “The local Colorado Springs Parkinson’s Group, we attend that on a regular basis, and we are members at large on their board. Of course, we’ve gathered the most strength from that.” Most participants reported caregiver support groups that focused on Parkinson’s disease were the most important for their continued success. Regarding the overall benefits of these groups for caregiving, Daisy indicated, “I think the most support we get is from the Parkinson’s support group.”

### Climbing the mountain

As caregivers, the participants were often overwhelmed by their husband’s illness but found ways to adapt and overcome their distress. Participants described how they pushed their needs and feelings aside to focus on their husband’s needs. These coping strategies sometimes resulted in increased tolerance for unwanted conduct by their husband but other times they learned walking away was easier. Lavender indicated, “I eventually had to quit working outside of the home because I was afraid for him. He was unstable and there’s stuff, circling. The vultures were circling, and I could recognize that, but I didn’t want to leave him alone.” Rose said, “My whole life is different, but then what’s the alternative? I pray for every day, another day, another day. Please keep him the same. I don’t want him getting worse.” Sadly, the problems eventually became so big it was hard to find solutions. Poppy admitted she began to drink more so she would feel less overwhelmed: “I’m drinking more. I would have a margarita with my friend in the pool, but now it’s like I just need something to take the edge off.” Lily addressed the lack of choice that made adaptation necessary: “You kind of don’t have a choice. You have to adapt your life to it. I mean, what else can you do, except run away.”

### Theme 3: not sure what to do next

During interviews, participants realized there was a specific point in their life when they fully appreciated their lives had changed. This change was reported as role changes or shifting from wife to caregiver, which resulted in subsequent changes in terms of control and power in the relationship. During the gradual advancement of the Parkinson’s disease, participants reported eventually being responsible for all the roles in the house. At that point, they finally recognized that they were dancing with their husbands to the music provided by the Parkinson’s disease. Lavender expressed this thought by saying “Because I am emotionally involved with this person [my husband], it’s like a fine dance. It’s a waltz. It’s between two partners. You have to learn how to trust, give, take, small pressures. It’s very nuanced in a relationship because there’s no clear delineation between responsibilities and how you interact with my husband.

### It’s my way now

Parkinson’s disease forced the participants to run the home. As Violet said, “If you look around us, whatever you see or imagine, the caregiver does.” This change of responsibilities encompassed all aspects of running a house and assuming responsibility for all the activities of daily living. Also, Iris highlighted all the responsibilities she managed because of her husband’s diagnosis:

I do all the bills and all the phone calls. Trim his fingernails, toenails, all that sort of thing. He used to be able to do just about anything around the house as far as electrical, or he could just do almost anything. And so now it’s hard to see that he really can’t do much at all because of the tremor. It’s just put more responsibility on me that I feel like I have to make a lot of the decisions.

The participants reported trying to leave some responsibilities for their husbands, even if it meant the task was performed more slowly or with assistance. Magnolia described sharing responsibilities in this way: “Making doctor’s appointments, I do that because he’s getting where he’s having trouble with word retrieval and saying the right words. So, I do that, but he still takes care of the books. He still does the banking stuff. It takes him a longer time, but he still does it.” Participants also indicated that their responsibilities were progressive. As the disease advanced, things slowly changed. Most participants attempted to discretely keep things working. Pansy described her attempts as follows:

I’m finding I’m doing a lot of picking up from him when he changes clothes. And taking care of things at night that he doesn’t take care of himself. And so that’s one of the things I’m doing all the time. I’m making his meals. He can’t do that. He is using a walker, trying to do more walking himself. I do all the driving.”

Adopting more responsibilities meant they also did more driving. Many participants discussed how they were always in the passenger seat before the Parkinson’s disease diagnosis. After the diagnosis, they were forced to drive. This transition was particularly difficult for the participants. Pansy said, “It was difficult at the beginning when I knew, when I got the sense that I was going to be the driver, I wasn’t going to be driven in the car. That was a little hard to think about that that’s the way life was going to change. So many things have changed that I just accept.” Almost all the participants eventually had to perform tasks they rarely did before the diagnosis, such as driving and managing the finances. This transition was a difficult experience for many of the participants. Having to do the driving seemed to be particularly stressful for most participants. Some common complaints included expressed by Violet and Iris were, “He would fall asleep while he was driving” and “I do all the driving.” Even when participants were talking about other responsibilities, they continued to mention driving. Poppy said, “It can be overwhelming. I’m doing the financial. He’s no longer driving; I do all the driving.” Similarly, Lily stated, “[I] totally manage his medications. I have had to take over driving 100%.”

### Nursing without a degree

Because caregiving became so pervasive in daily life, participants eventually provided their husbands with the same level of care as a nurse. Lavender described the distinction between being a spouse and a nurse in the following way:

How am I supposed to put my nurse hat on, take my nurse hat off? It’s not one or the other. It’s blending it constantly. And sometimes you have to add a little more butter or add a little more salt. Every day is a dance that you have to do.

As the disease progressed, participants needed to manage the activities of daily living, such as bathing and feeding. They also reported they were even monitoring their husbands during the night. Poppy said, “You listen all night long. Is he breathing? Is he breathing? I curl up to him and rock him, and that usually gets him to breathe again. So, you’re not really getting good sleep because you’re making sure they’re still alive all night long.”

With the increased caregiving responsibilities, the participants became more dedicated to making sure their husbands were safe. As Pansy stated, “I really feel like I don’t have any time on my own. I have to be aware of where he is because he has started falling. I have to be aware to make sure that he’s using his walker, where he is going, how long he’s been there, do I need to go check on him?” Some participants indicated their 24-hour, seven-days-a-week responsibility as a caregiver was like being on call. Lilac said, “I feel like I have taken the attitude of a doctor on call. And it’s a 24/7 thing because it might be the middle of the night. Like he might suddenly realize his mask might not fit him well and have to adjust it or whatever because his hands don’t work for Parkinson’s.”

### Emotional rollercoaster

The participants reported each day was an ongoing emotional struggle that began when they woke up in the morning and lasted until they went to bed late at night. Most participants spoke about alternating emotions that swung from extreme highs to lower lows. Many participants described the process as a rollercoaster because they never really knew how the day would go from minute to minute. Lavender felt that “I don’t deserve to be spoken to or treated that way because whatever you’re experiencing. So, I know you have enough self-control, so I’m happy to put myself in time out and go walk on the beach. It’s not always ‘Okay, I see the end’ because you can’t see the end.” Other participants described how adjusting their way of life was difficult but also a compromise. Specifically, Petunia said the following:

Well, it’s certainly been an adjustment that we did not expect to make, but yet going into a marriage, you know you grow old over time, and you know something’s going to happen with one if not both of you. So, you just have to try to deal with it as it comes along. He should be able to lead a fairly good quality of life. The better his quality of life is, the better mine will be.

At times, the participants described their role as that of the mother of the household rather than the wife. Rose stated, “Listen, I now wipe his tush when he has a bowel movement. When I say I’m the mother, I’m not kidding.” Most participants described this role shift in terms of needing to help their husband remember basic things or how to do different tasks. Iris said, “It’s hard to not be around when I leave. I always make sure he’s got his phone on because if he happened to fall or something.” Other participants provided their husbands with detailed reminders about tasks in the same way as they once reminded their children. Carnation these types of reminders as follows:

I really have to watch him because sometimes he puts dirty clothes in the dresser drawer and then sometimes, he’ll pull out clean things and put them in the dirty clothes or he’ll try to put on two pair of sleeping pants, that kind of thing. It’s like the brain is almost frozen. And he just can’t process the everyday activities. It’s living with a little child that you are totally responsible for what goes on.

Some participants indicated they tried to continue with normal activities, such as socializing with friends, but realized they needed to arrange extra care for their husband as they once did for their children. Poppy expressed this rollercoaster of lack of socialization with friends and extra care as follows:

Are you kidding me? So really you have no life but what you make of your own, and the socialization with the friends that we have, they have been beautiful, beautiful people to have met. Very fortunate in that. So daily life is what am I making for dinner? Is he dressed? He can get the shirt over his head, but he can’t pull it down on the back. So, you’re helping him dress. Reminding him, “Take your inhaler. Take your Flonase. Did you brush your teeth before you came to bed?”

### Theme 4: just too much

As participants assumed the new role of being a caregiver, they indicated a feeling of preoccupation with the new direction of their life. For instance, emotions were difficult to control, more spontaneous with time, as their life changed beyond recognition. As such, participants had to decide how to manage physical and mental situations, which often resulted in feelings of anger. Participants frequently wondered how they could manage the craziness that had become their life. Carnation expressed these emotions and anger in the following recollection:

Probably the worst thing that really set me off about a month ago was he had been showering in one bathroom. I was getting cleaned up in another one. And when I went out into the kitchen, I opened up a drawer that has nice spatulas, fairly deep drawer. And there was a wet Depends diaper. And that really set me off. That was almost more than I could take. And of course, he said he didn’t do it.

### Physical and mental overload

People with Parkinson’s disease experience multiple challenges and limitations that must be consistently addressed. They may have limitations with eating or getting dressed, they may have cognitive decline from brain fog or dementia, or they may have mechanical challenges with walking, eating, and bathing. As caregivers, the participants had to handle these physical challenges and limitations for their husbands. Poppy expressed this frustration with, “When they told us there would be some cognitive decline, we did not realize the amount of cognitive decline.” Participants indicated early mornings were exceptionally difficult. Another woman, Carnation, said, “He wakes up and he may be confused or start just talking about something that doesn’t make sense. And so, that’s changed life a lot because we used to go a lot, do a lot of things, and can’t do that anymore. That’s just part of where we are in with this illness.” Other participants told stories of how cognitive challenges clouded their entire day, not just the early mornings. As Crocus said, “Losing some of the sharp edge, whether that’s just ageing in general or some beginning of some dementia, I’m not in a place where I can diagnose that, but we’re noticing differences.”

Cognitive challenges were not the only issues participants encountered. They also described their husband’s physical challenges like tremors and freezing in place when trying to walk. Examples of this behaviour were expressed as the following: “His tremor, it’s worse in the mornings. He has trouble getting his coffee. And so, I guess that I think that would be the most challenging” (Iris). Lilac said, “The other hardest thing right now is just this freezing, this sort of skittering steps that he takes,” but she added, “Now he can hardly speak to be understood.” To help their husbands, participants tried to talk them through problems, using conversation and encouragement. As Daisy explained, “He started shuffling his feet, and I’m always reminding him, ‘Come on, big steps, not these baby steps, because that’s not good.’ And he goes, ‘I’m working on it; I’m working on it.’” Some participants found they needed to physically touch their husbands to help recalibrate their mind. As conveyed by Magnolia, touch could be especially therapeutic:

I can touch him and make him aware of another part of his body. It kind of wakes up the brain, I think, and he’ll stop, and then the next step he takes is a good step. But if I’m not close enough to touch him, I try and say, “honey, stop.” And if he looks at me, and I say stop again, again it’s like he’s activating that brain again to rethink what his legs are doing. I don’t know if that makes sense.

### I’m just so angry

Because the participants had put their lives on hold to take care of their husbands, many felt anger. Sometimes their frustration was directed towards their spouse, and other times it was directed towards the entire gravity of the situation. Lilac expressed this anger by stating, “So I struggle with my own, like, reactions of anger. Like, how could you not hear that or understand that, or I just said that to you. I have to repeat myself a lot.” The participants also described having an internal struggle with their anger and frustration, but they had no way to talk it through it with their spouses. As Carnation said, “Well, sometimes I just have to bite my lip and turn the cheek and do something else. Walk away from it because if he gets upset or angry about something you can’t push on it. It’s not like you can talk it out.” Pansy started to cry when asked this question. She felt discouraged about the amount of isolation associated with this disease, but she accepted it because she had to. She also indicated that she struggled to maintain her social life even though he was unwilling to participate in life, which caused her more grief, anger, and stress. Pansy described this experience as follows:

I’m noticing when I go out, he wants to know every place I’ve been. And sometimes I don’t want to tell him, or I’m just ordinary [in the] things I was doing, errands. Just having friends, it’s a struggle to even have friends over anymore. That’s hard for me because I like to serve. I like to have dinners and enjoy company with people, and he’s not so much anymore. And he is on a diet, so eating out isn’t what he can do that much. And that’s okay with me. I like cooking, so I don’t mind eating at home. But probably the hardest part is not wanting to be with friends.

### Managing the craziness

As daily caregivers, the participants figured out how to deal with their stress, burdens, and anxiety and depression. To manage these feelings and the craziness of life, participants indicated that they considered some days for themselves and other days for their spouse. Rose said, “On bad days, I’m a bitch. I’m angry, and I’m resentful, and it’s not his fault. Then I become guilty. Then at night, I’ll start to cry and apologize because it’s not his fault. He took care of me; it’s now my turn. I know that intellectually.” Many of the participants said they relied on their faith and often asked for help through prayer and blessings. For example, Violet stated, “Well, like I said, it’s very faith based. If I need help, I can ask for blessings,” and Magnolia said, “I pray. I just give it to the Lord, and I take it off my shoulders. That’s it.” Other participants used their friends or outside activities to deal with the monotony of their situations. Doing so helped break the cycle and gave the caregivers a moment to breathe. Petunia detailed how planned activities with friends helped things seem more normal:

So, we’re going out to a restaurant with everyone tonight, and then they’re coming to our house for dessert, and we’ll play a game or something. We try to do that. Every Thursday, we try to set it up with some friends. We play a specific game called Mexican Train. Just anything like that that will settle things down for a little while.

### Theme 5: caring is your soul’s growth

“Caring is your Soul’s growth” was a comment made by Lilac when discussing her experiences of caring for her husband. In general, the participants explained the slow disease progression in the context of their spiritual growth. Participants gradually increased their role as caregiver, which allowed time for reflection, essential to continue growing as a new person. Lilac explained this growth as follows:

One way that I looked at this is, and is outside of the kind of normal medical thing, but it’s more of a spiritual thing that has helped me to say, okay, what if on a spiritual level, you signed up as a contract for your life to get some, that this is part of your soul’s growth, that by helping this other person, by sacrificing and letting go of the things that you have to no longer be able to do with him together, or even dreams that I might have had for us as a couple that we can’t do anymore.

### Please, God, help me

Participants frequently declared a need to seek God’s assistance to get through the process. As Carnation stated, “I have God on my side, so I have family and friends who include me in their prayers.” Others described their spirituality and how they were able to find solutions from within. Lavender declared, “As a spirit, I believe my purpose is to be of service and that I am intelligent and can figure out. I can find a solution to all things.” Some participants believed in their wedding vows and took the “for better or worse” part very seriously. As such, they were very dedicated to their marriage through God’s assistance. Pansy expressed this idea as follows:

I am a Christian, and I have promised when I married him to love him no matter what until we’re both gone or I’m gone right, or he’s gone. And I am dedicated to that knowing that God is with me and helping me. I don’t know if someone didn’t have God in their life how they would handle it.

### Finding my inner strength

Because of their commitment to their spouses, participants had an overwhelming drive to do what was genuine to uphold their marital commitment. This commitment required resilience, expressed through an inner strength. Lily described her commitment by saying, “I am really good at helping other people when it’s not me. I guess the only strength that I can really pull from is the fact I understand what’s happening because I’ve been there before.” They also embraced the journey while digging for the strength to move forward. As Rose said, “I’m gentle but strong when I have to be, but I’m stronger than a bull. There are times when my mind goes to maybe I shouldn’t have married him. When it boils down to it, I say to myself, ‘Would you rather have had somebody else?’ No, he’s, my husband. I love him.” Crocus stated, “This is just something I need to do.” As observed so many times, most participants acknowledged that a great deal of their inner strength arose from their faith. Iris commented, “I think my faith, being a Christian, is the biggest thing that gives me inner strength. And friends. Just a lot of support from friends. It’s part of life, and that he’d be there for me, and I want to be there for him. And I guess it’s just a commitment and a strong faith.”

At times, participants began to realize the problem was bigger than they were and keeping busy helped them stay focused. As Magnolia stated, “I’m able to focus on something else other than his problems. And I think just being active, and keeping busy, and keeping my mind busy, and doing other things helps me.” As participants discussed being a caregiver, they realized that, although they were driven to care for their spouses, they were learning along the way. Lilac said, “Having to care for him has made me a much better person than I would have been. I’m kinder. I’m more compassionate. I’m more tolerant. I’m more patient. I’m more flexible. I’m less perfectionist and demanding.” Poppy stated, “I want to continue sharing all the life experiences we can together and make sure he realizes he’s still worthy. So, that’s what drives me. I love him.” Some women found that humour helped them get through the journey. Daisy recounted such interactions in the following:

A bit of humor. He says, “Just call me Abby, Abby Normal.” So, he used his sense of humor to get through things. And some days, I’ll just look at him, and I’ll go, “This is truly an Abby day.” And he goes, “Yes, it is. That’s the way I am.” I think I’m still the same person. Little older, but I still have friends, I still make connections.

### How can I help you?

Participants frequently pursued self-education to learn more about Parkinson’s disease and overcome obstacles associated with the illness. Because they took this initiative, participants were exceedingly happy to offer advice to future caregivers about how to obtain information and find what they needed to know to move forward. Carnation said, “Look for all the info that you can find as far as the disease is [concerned]. Look for all the support that you can find.” The participants also offered words of encouragement based on their experiences, as expressed by Pansy in the following:

I would encourage anyone having the experience of a husband or wife with Parkinson’s to learn as much as you can about it and the causes … . Experiences that they’ll go through so that you’re not unaware of that and attend as many meetings as you can in your area to have support from the group. [Colorado Parkinson’s Foundation]

Participants suggested that new caregivers should research the illness to learn about it so they could better understand what caring for a person with Parkinson’s disease would look like. Magnolia suggested the following:

I would say when they first learn that they’re going to be involved with caregiving I think if they get involved with some kind of a group that has the knowledge and resources to support them in whatever they’re going to need would be an important factor for them to consider. And just talking to other people who are in the know about what Parkinson’s is going to look like as their person progresses, and just being able to know, “Okay, this might happen. This might happen.”

In same way, participants described a community of caregivers as a valuable resource. They also explained that, as the community begins to age, there will be a bigger need for individuals to offer caregiving services to their families. Crocus stressed this need by saying, “There are more caregivers out there, which is, I suppose, partly in response to the ageing general population and the fact that we have much greater need for more caregivers, that people are willing to do that, people who hadn’t considered that before, that they’re considering it as a career.”

## Discussion

The purpose of the current study was to describe the lived experiences of women caregivers of male spouses living with Parkinson’s disease. Using a phenomenological design and interviews, participants provided detailed descriptions about their experiences, including information about obstacles and emotions and how they navigated life. As a result, rich details emerged from the interviews about the lived experience of the wife caregivers.

After analysis, a central theme of the lived experiences of wives caring for husbands living with Parkinson’s disease was “There is no cure for caregiving” as the caregivers were unable to continue being wives. The findings from this study were largely congruent with research (Prado et al., 2020; Zucca et al., 2021) investigating other neurodegenerative and neurocognitive diseases. Specifically, women caregivers had to contend with emotional, financial, and physical burdens that originated from their husband’s Parkinson’s disease diagnosis (Boersma et al., 2017). Further, these burdens became more difficult as the disease advanced through the Hoehn and Yahr stages (Modestino et al., 2018). For this reason, self-monitoring is an effective strategy for motivating self-management when the caregiver and the patient are included in team discussions with the physician about medical care (Hellqvist et al., 2020).

As the primary caregiver for their husband, the wives managed all aspects of life, including the basic activities of daily living. Previous studies have reported similar roles (Prado et al., 2020). Most participants in the current study reported that they experienced conflicting emotions after their husband’s Parkinson’s disease diagnosis. They expressed the idea of slowly disappearing from life and fading into the background as the disease emerged in the foreground. They also realized that they are the essential primary health care providers and are responsible for keeping their husbands at home as the disease progresses. Even though the majority of people living with long-term debilitating conditions such as Parkinson’s disease rely heavily on caregivers, most physicians have little knowledge about when to respond to caregiver concerns or how to best address their needs (Schwartz et al., 2020).

Physicians, usually neurologists or movement disorder specialists, are in a unique position to diagnose, medically manage, and collaborate with women caregivers of husbands living with Parkinson’s disease (Willis et al., 2011). Although their primary focus is medical management, physicians need to recognize the importance of caregivers and acknowledge them as a part of the health care team. Unfortunately, little research has investigated physician understanding about caregiving or the experiences of caregivers (Dekawaty et al., 2019). The caregivers in the current study wanted to be recognized as members of the health care team and as wives of men living with Parkinson’s disease. For this reason, the participants wanted more information about resources that would help them become better caregivers and manage the complexity of their new role as caregivers.

Retirement is typically viewed as a rite of passage that allows people to spend more time with family and friends. During these years, most couples want to disengage from full-time work responsibilities and focus on “doing what makes you happy” (Savishinsky, 2002). Although the women of the current study wanted to enjoy retired life through relaxation, spontaneity, and leisurely activities, their husband’s Parkinson’s disease diagnosis occurred about the same time and shattered that retirement dream. The women continued to work day by day in a new role often wondering who would help them as the work became increasingly strenuous and more consuming. As explained by self-determination theory (Ryan & Deci, 2000), the women eventually lost the sense of who they were and, in some cases, the ability to relate to their husband’s condition.

Anger, guilt, and resentment were common emotions described by most participants in the current study. Importantly, the negative emotions were not necessarily directed towards their spouses so much as at the situation. Although conflicting emotions are normal for caregivers (Rosqvist et al., 2022), these emotions are typically suppressed by wives out of a moral obligation to care for their husbands (Kleinman, 2013). As a result, wives increasingly feel overwhelmed with caregiving until they are emotionally exhausted (Kontrimiene et al., 2021). A study by Bhasin and Bharadwaj (2021) suggested that wife caregivers succumb to their “implicit commitment of bearing with unending uncertainty, a restricted sense of autonomy and a silent acquiescence of being self-sacrificial” (p. 8). The women of the current study reported lacking the capacity to manage stressful situations and had difficulty managing emotions, which often resulted in feelings of anger. Yet, they also reported finding an inner strength that helped them continue moving forward. Usually, they stated that their religious faith and spiritual beliefs supported their inner strength. At that point in the disease process, similar to other research (Habermann et al., 2013), the wives experienced personal growth, shifting from self-centeredness to being more caring.

Most participants in this study needed external support for household activities and moments of respite from caring for their husbands. Caregiver identity theory explains why women need a network of family members, close friends, and support groups (Montgomery & Kosloski, 2009). Without social support early in the caregiving trajectory (Dunk et al., 2017), women may experience caregiver burden, emotional exhaustion, and burnout, particularly when these issues are left unattended (Gultekin et al., 2017; Kang et al., 2020). The women in the current study found solace in family relationships and benefits from support groups that allowed them to connect with other women who understood their situation. These outlets were not enough for some, however. Many women admitted to increased alcohol consumption to escape the realities of their new life as a caregiver. Similar to previous research (Kumar et al., 2022), increased responsibilities, financial strain, and disruptions in daily living made these caregivers more vulnerable to alcohol abuse.

A majority of participants described being awake most of the time because they were providing direct care for their spouses. They assisted their husbands with bathing, feeding, and other activities. Over time and as the disease progressed, these loving wives and equal partners in the spousal relationship had their role shift to full-time caregivers with parental responsibility for managing the household. Similar to other studies (Supaporn et al., 2022), these increased medical demands resulted in the women of the current study becoming uncompensated nursing assistants who were responsible for feeding, bathing, and medicating their spouses. The theory of human caring (Watson, 2012), which originated in nursing (Watson, 1999), defines carative factors as caring relationships, caring moments, authentic presence, and acceptance of negative and positive feelings (Alharbi & Baker, 2020). When considering these factors in relation to study results, this theory may explain the caring sentiments reported by study participants. These women struggled to make their husbands feel safe, cared for, and cared about despite their inner conflicts with various emotions.

As the Parkinson’s disease progressed over time, the women became the primary drivers of everything in the household from driving the car to medical appointments to making all financial decisions. Before their husband’s Parkinson’s diagnosis, the women were accustomed to sitting in the passenger seat. Eventually, the wives were forced to drive because their husbands were no longer capable of it. For the women of this study, driving represented an ongoing psychological obligation that was incredibly distressing. Even though our participants highlighted this issue during interviews, to our knowledge, previous research has not investigated this aspect of caregiver burden. The women were forced to drive, even though they disliked it, because they needed to be mobile and feared their spouses would be harmed if they attempted to drive (Berger et al., 2019). More research is needed to determine if this situation is generational or generalizable to the wife caregivers of people living with similar diseases.

Over time, the participants needed to engage in new activities and accept more household responsibilities. As caregiving requires increasingly more time with disease progression, the women had less time available to continue doing the things they personally enjoyed. In the end, the participants continued to experience feelings of anger, fear, and resentment.

### Limitations

The current study had some limitations to report. First, study participants were recruited from a single organization located in Colorado Springs, Colorado, limiting the generalizability of findings. Due to the possibility of identifying participants, some sociodemographic information was not disclosed. With this stated, more research is necessary to understand the caregiving experiences of wives from different racial and ethnic groups. The participants in this study were already participating in a support group. However, information about participant participation in other support groups was not collected. The current study did not include the lived experiences of spouses to obtain a deeper understanding about the impact of Parkinson’s disease on the caregiver as part of a couple. Future research should study the lived experience of spouse caregiver and patient to better understand the dynamic of Parkinson’s disease, the couple relationship, and caregiving. Older adults uncomfortable with technology may have been interested in participating in the study but unwilling or unable to use Zoom. However, one woman who did not have a computer participated in the study with the assistance of staff from the Colorado Parkinson Foundation. Despite these limitations, the current study provides important insights into the lived experience of women as caregivers of male spouses with Parkinson’s disease.

## Conclusions

Wife caregivers of husbands living with Parkinson’s disease at home provide basic health care services, run the household, manage the finances, and organize medical appointments. As they assume more responsibility for managing their husband’s health, the caregivers feel increasingly invisible to the health care team. Wife caregivers passively seek to be acknowledged as part of the team rather than identified as the wife of a patient. As such, clinical professionals need to be authentically present for the wife caregiver as they medically manage their mutual patient’s disease. Due to the limited time to address caregiver burden during routine office visits, particularly when patients are very sick, clinicians should be mindful of helping caregivers access additional resources such as social workers and palliative care services. Furthermore, caregiver support groups, education programmes, and telemental health services are important strategies to reduce caregiver burden, limit occupational injuries, and prevent emotional exhaustion. Policy makers should formally recognize the contribution of wife caregivers through funded initiatives and reimbursement models as essential resources for preventing the expensive premature institutionalization of people living with Parkinson’s disease.

## Supplementary Material

IJQSHW_SUPPFILE01_20230523_v1_FINAL.docx

## Data Availability

The data used and analysed during the current study are available from the corresponding author on reasonable request. As the qualitative data resulted from interviews, the complete transcripts will not be released without the written consent of each participant. However, an expanded data file is provided in a supplemental table with additional quotes to support the analysis.

## References

[cit0001] Alase, A. (2017). The interpretative phenomenological analysis (IPA): A guide to a good qualitative research approach. *International Journal of Education & Literacy Studies*, 5(2), 9. 10.7575/aiac.ijels.v.5n.2p.9

[cit0002] Alharbi, K., & Baker, D. O. G. (2020). Jean Watson’s middle range theory of human caring: A critique. *International Journal of Advanced Multidisciplinary Scientific Research*, 3(1), 1–19. 10.31426/ijamsr.2020.3.1.3011

[cit0003] Archibald, M. M., Ambagtsheer, R. C., Casey, M. G., & Lawless, M. (2019). Using zoom videoconferencing for qualitative data collection: Perceptions and experiences of researchers and participants. *International Journal of Qualitative Methods*, 18, 1609406919874596. 10.1177/1609406919874596

[cit0004] ATLAS.ti Scientific Software Development GmbH. Atlas.Ti 22 for windows 2022. Available from: https://atlasti.com

[cit0005] Balash, Y., Korczyn, A. D., Migirov, A. A., & Gurevich, T. (2019). Quality of life in Parkinson’s disease: A gender-specific perspective. *Acta Neurologica Scandinavica*, 140(1), 17–22. 10.1111/ane.13095 30953570

[cit0006] Baldereschi, M., DiCarlo, A., Rocca, W. A., Vanni, P., Maggi, S., Perissinotto, E., Grigoletto, F., Amaducci, L., & Inzitari, D. (2000). Parkinson’s disease and parkinsonism in a longitudinal study: Two-fold higher incidence in men. *Neurology*, 55(9), 1358–1363. 10.1212/WNL.55.9.1358 11087781

[cit0007] Barriball, K. L., & While, A. (1994). Collecting data using a semi-structured interview: A discussion paper. *Journal of Advanced Nursing*, 19(2), 328–335. 10.1111/j.1365-2648.1994.tb01088.x 8188965

[cit0008] Berger, S., Chen, T., Eldridge, J., Thomas, C. A., Habermann, B., & Tickle-Degnen, L. (2019). The self-management balancing act of spousal care partners in the case of Parkinson’s disease. *Disability and Rehabilitation*, 41(8), 887–895. 10.1080/09638288.2017.1413427 29228835 PMC6179928

[cit0009] Bhasin, S. K., & Bharadwaj, I. U. (2021). Perceptions and meanings of living with Parkinson’s disease: An account of caregivers lived experiences. *International Journal of Qualitative Studies on Health and Well-Being*, 16(1), 1967263. 10.1080/17482631.2021.1967263 34414851 PMC8381973

[cit0010] Biderman, A., Carmel, S., Amar, S., & Bachner, Y. G. (2021). Care for caregivers- a mission for primary care. *BMC Family Practice*, 22(1). 10.1186/s12875-021-01579-6 PMC8593856 34784890

[cit0011] Boersma, I., Jones, J., Coughlan, C., Carter, J., Bekelman, D., Miyasaki, J., Kutner, J., & Kluger, B. (2017). Palliative care and Parkinson’s disease: Caregiver perspectives. *Journal of Palliative Medicine*, 20(9), 930–938. 10.1089/jpm.2016.0325 28520498 PMC5576067

[cit0012] Cao, F., Souders Ii, C. L., Perez-Rodriguez, V., & Martyniuk, C. J. (2019). Elucidating conserved transcriptional networks underlying pesticide exposure and Parkinson’s disease: A focus on chemicals of epidemiological relevance. *Frontiers in Genetics*, 9, 701. 10.3389/fgene.2018.00701 30740124 PMC6355689

[cit0013] Cerri, S., Mus, L., & Blandini, F. (2019). Parkinson’s disease in women and men: What’s the difference? *Journal of Parkinson’s Disease*, 9, 501–515. 10.3233/JPD-191683 PMC6700650 31282427

[cit0014] Champagne, E. R., & Muise, A. (2022). Responsiveness and relationship satisfaction in couples coping with Parkinson’s disease: A pilot study. *Psychological Reports*, 125(2), 804–821. 10.1177/0033294121998032 33626979 PMC9003747

[cit0015] Cianfrocca, C., Caponnetto, V., Donati, D., DiStasio, E., Tartaglini, D., & Lancia, L. (2020). The opinions and feelings about their educational needs and role of familial caregivers of Parkinson’s disease patients: A qualitative study. *Acta Bio-Medica: Atenei Parmensis*, 91(12–S), e2020002. 10.23750/abm.v91i12-S.10264 PMC8023112 33263347

[cit0016] Colaizzi, P. F. (1973). *Reflection and research in psychology*. Kendall Hunt.

[cit0017] Colaizzi, P. F. (1978). Psychological research as a phenomenologist views it. In R. Valle & M. King (Eds.), *Existential phenomenological alternatives for psychology* (pp. 48–71). Open University Press.

[cit0018] Dahodwala, N., Shah, K., He, Y., Wu, S. S., Schmidt, P., Cubillos, F., & Willis, A. W. (2018). Sex disparities in access to caregiving in Parkinson disease. *Neurology*, 90(1), e48–e54. 10.1212/WNL.0000000000004764 29196580 PMC10681055

[cit0019] Dekawaty, A., Malini, H., & Fernandes, F. (2019). Family experiences as a caregiver for patients with Parkinson’s disease: A qualitative study. *Journal of Research in Nursing*, 24(5), 317–327. 10.1177/1744987118816361 34394542 PMC7932426

[cit0020] Dorsey, E. R., Elbaz, A., Nichols, E., Abbasi, N., Abd-Allah, F., Abdelalim, A., Adsuar, J. C., Ansha, M. G., Brayne, C., Choi, J. Y. J., Collado-Mateo, D., Dahodwala, N., Do, H. P., Edessa, D., Endres, M., Fereshtehnejad, S.-M., Foreman, K. J., Gankpe, F. G. … Vos, T. (2018). Global, regional, and national burden of Parkinson’s disease, 1990–2016: A systematic analysis for the global burden of disease study 2016. *The Lancet Neurology*, 17(11), 939–953. 10.1016/S1474-4422(18)30295-3 30287051 PMC6191528

[cit0021] Dorsey, E. R., Sherer, T., Okun, M. S., Bloem, B. R., Brundin, P., Langston, J. W., & Bloem, B. R. (2018). The emerging evidence of the Parkinson pandemic. *Journal of Parkinson’s Disease*, 8(s1), S3–S8. 10.3233/JPD-181474 PMC6311367 30584159

[cit0022] Dunk, M., Engblom, H., Gissen, M., Joseph, E., Po-Fai Li, J., Russell, D. W., & McRae, C. (2017). Social support and loneliness among Parkinson care partners. *Medical Research Archives*, 5(8), 1–14.

[cit0023] Elo, S., Kääriäinen, M., Kanste, O., Pölkki, T., Utriainen, K., & Kyngäs, H. (2014). Qualitative content analysis. *SAGE Open*, 4(1), 215824401452263. 10.1177/2158244014522633

[cit0024] Fang, C., Lv, L., Mao, S., Dong, H., & Liu, B. (2020). Cognition deficits in Parkinson’s disease: Mechanisms and treatment. *Parkinson’s Disease*, 2020, 2076942. 10.1155/2020/2076942 PMC7128056 32269747

[cit0025] Funayama, M., Nishioka, K., Li, Y., & Hattori, N. (2023). Molecular genetics of Parkinson’s disease: Contributions and global trends. *Journal of Human Genetics*, 68(3), 125–130. 10.1038/s10038-022-01058-5 35821405 PMC9968657

[cit0026] Geerlings, A. D., Kapelle, W. M., Sederel, C. J., Tenison, E., Wijngaards-Berenbroek, H., Meinders, M. J., Munneke, M., Ben-Shlomo, Y., Bloem, B. R., & Darweesh, S. K. L. (2023). Caregiver burden in Parkinson’s disease: A mixed-methods study. *BMC Medicine*, 21(1), 247. 10.1186/s12916-023-02933-4 37424022 PMC10332089

[cit0027] Gentles, S. J., Charles, C., Ploeg, J., & McKibbon, K. (2015). Sampling in qualitative research: Insights from an overview of the methods literature. *The Qualitative Report*, 20(11), 1772–1789. 10.46743/2160-3715/2015.2373

[cit0028] Gray, L. M., Wong-Wylie, G., Rempel, G. R., & Cook, K. (2020). Expanding qualitative research interviewing strategies: Zoom video communications. *The Qualitative Report*, 25(5), 1292–1301. 10.46743/2160-3715/2020.4212

[cit0029] Guba, E. G., & Lincoln, Y. S. (1994). Competing paradigms in qualitative research. In N. Denzin & Y. Lincoln (Eds.), *Handbook of qualitative research* (pp. 105–117). Sage Publishing.

[cit0030] Guest, G., Namey, E., Chen, M., & Soundy, A. (2020). A simple method to assess and report thematic saturation in qualitative research. *PLOS*, 15(5), e0232076. 10.1371/journal.pone.0232076 PMC7200005 32369511

[cit0031] Gultekin, M., Ekinci, A., Erturk, G., & Mirza, M. (2017). Female Parkinson’s disease caregivers have much anxiety and depressive symptom. *Brain and Behavior*, 7(9), e00787. 10.1002/brb3.787 28948082 PMC5607551

[cit0032] Habermann, B., Hines, D., & Davis, L. (2013). Caring for parents with neurodegenerative disease. *Clinical Nurse Specialist*, 27(4), 182–187. 10.1097/NUR.0b013e318295576b 23748990 PMC3682660

[cit0033] Hand, A., Oates, L. L., Gray, W. K., & Walker, R. W. (2019). The role and profile of the informal carer in meeting the needs of people with advancing Parkinson’s disease. *Aging & Mental Health*, 23(3), 337–344. 10.1080/13607863.2017.1421612 29293027

[cit0034] Hellqvist, C., Berterö, C., Hagell, P., Dizdar, N., & Sund-Levander, M. (2020). Effects of self-management education for persons with Parkinson’s disease and their care partners: A qualitative observational study in clinical care. *Nursing & Health Sciences*, 22(3), 741–748. 10.1111/nhs.12721 32270898

[cit0035] Hoehn, M. M., & Yahr, M. D. (1967). Parkinsonism onset, progression, and mortality. *Neurology*, 17(5), 427–427. 10.1212/WNL.17.5.427 6067254

[cit0036] Hoogland, J., Post, B., & de Bie, R. M. A. (2019). Overall and disease related mortality in Parkinson’s disease – A longitudinal cohort study. *Journal of Parkinson’s disease*, 9, 767–774. 10.3233/JPD-191652 PMC6839485 31498129

[cit0037] Husserl, E. (2012). *Ideas: General introduction to pure phenomenology*. Routledge.

[cit0038] Iwasa, Y., Saito, I., & Suzuki, M. (2021). Differences in home health nursing care for patients with Parkinson’s disease by stage of progress: Patients in Hoehn and Yahr stages III, IV, and V. *Parkinson’s Disease*, 2021, 8834998. 10.1155/2021/8834998 PMC7920702 33688425

[cit0039] James, S. L., Abate, D., Abate, K. H., Abay, S. M., Abbafati, C., Abbasi, N., Abbastabar, H., Abd-Allah, F., Abdela, J., Abdelalim, A., Abdollahpour, I., Abdulkader, R. S., Abebe, Z., Abera, S. F., Abil, O. Z., Abraha, H. N., Abu-Raddad, L. J., Abu-Rmeileh, N. M. E., Accrombessi, M. M. K., Acharya, D., Vos, T. (2018). Global, regional, and national incidence, prevalence, and years lived with disability for 354 diseases and injuries for 195 countries and territories, 1990–2017: A systematic analysis for the global burden of disease study 2017. *The Lancet*, 392(10159), 1789–1858. 10.1016/S0140-6736(18)32279-7 PMC6227754 30496104

[cit0040] Juneja, A., Anand, K., Chandra, M., Deshpande, S., Dhamija, R., Kathuria, P., & Mahajan, R. (2020). Neuropsychiatric symptoms and caregiver burden in Parkinson’s disease. *Annals of Indian Academy of Neurology*, 23(5), 656–660. 10.4103/aian.AIAN_91_20 33623267 PMC7887493

[cit0041] Kang, S. G., Song, S. W., Kim, S. H., Kang, Y. J., Kim, Y. R., & Eun, Y. (2020). Fatigue and mental status of caregivers of severely chronically ill patients. *Pain Research & Management*, 2020, 6372857. 10.1155/2020/6372857 32963657 PMC7492882

[cit0042] Kist Bakof, K., Morais Machado, L., Rocha Iensen, G., Iwersen Faria, S., Silva Rodrigues, I., Passaglia Schuch, A., Jacques Schuch, N., & Boeck, C. R. (2021). Stress and its contribution to the development of depression symptoms are reduced in caregivers of elderly with higher educational level. *Stress*, 24(6), 676–685. 10.1080/10253890.2021.1876659 33461390

[cit0043] Kleinman, A. (2013). From illness as culture to caregiving as moral experience. *New England Journal of Medicine*, 368(15), 1376–1377. 10.1056/NEJMp1300678 23574117

[cit0044] Klietz, M., von Eichel, H., Schnur, T., Staege, S., Hoglinger, G. U., Wegner, F., & Stiel, S. (2021). One year trajectory of caregiver burden in Parkinson’s disease and analysis of gender-specific aspects. *Pain and Research Management*, 11(3), 295. 10.3390/brainsci11030295 PMC7996933 33652825

[cit0045] Kontrimiene, A., Sauseriene, J., Blazeviciene, A., Raila, G., & Jaruseviciene, L. (2021). Qualitative research of informal caregivers’ personal experiences caring for older adults with dementia in Lithuania. *International Journal of Mental Health Systems*, 15(1), 12. 10.1186/s13033-020-00428-w 33472676 PMC7816390

[cit0046] Krouwel, M., Jolly, K., & Greenfield, S. (2019). Comparing Skype (video calling) and in-person qualitative interview modes in a study of people with irritable bowel syndrome – an exploratory comparative analysis. *BMC Medical Research Methodology*, 19(1), 219. 10.1186/s12874-019-0867-9 31783797 PMC6883529

[cit0047] Kumar, S., Schess, J., Velleman, R., & Nadkarni, A. (2022). Stigma towards dependent drinking and its role on caregiving burden: A qualitative study from Goa, India. *Drug and Alcohol Review*, 41(4), 778–786. 10.1111/dar.13438 35128746 PMC9304139

[cit0048] Lennaerts-Kats, H., Ebenau, A., Steppe, M., van der Steen, J. T., Meinders, M. J., Vissers, K., Munneke, M., Groot, M., & Bloem, B. R. (2020). “How long can I carry on?” the need for palliative care in Parkinson’s disease: A qualitative study from the perspective of bereaved family caregivers. *Journal of Parkinson's Disease*, 10(4), 1631–1642. 10.3233/JPD-191884 PMC8764597 32651330

[cit0049] Lincoln, Y. S., & Guba, E. G. (1985). *Naturalistic inquiry*. Sage Publication.

[cit0050] Lobe, B. (2017). Best practices for synchronous online focus groups. In R. Barbour & D. Morgan (Eds.), *A new era in focus group research: Challenges, innovation and practice* (pp. 227–250). Palgrave Macmillan.

[cit0051] Lobe, B., Morgan, D., & Hoffman, K. A. (2020). Qualitative data collection in an era of social distancing. *International Journal of Qualitative Methods*, 19, 1609406920937875. 10.1177/1609406920937875

[cit0052] Lopez, K. A., & Willis, D. G. (2004). Descriptive versus interpretive phenomenology: Their contributions to nursing knowledge. *Qualitative Health Research*, 14(5), 726–735. 10.1177/1049732304263638 15107174

[cit0053] Marras, C., Beck, J. C., Bower, J. H., Roberts, E., Ritz, B., Ross, G. W., Abbott, R. D., Savica, R., Van Den Eeden, S. K., Willis, A. W., & Tanner, C. M. (2018). Prevalence of Parkinson’s disease across North America. *NPJ Parkinson’s Disease*, 4(1), 21. 10.1038/s41531-018-0058-0 PMC6039505 30003140

[cit0054] Martinez-Martin, P., Skorvanek, M., Henriksen, T., Lindvall, S., Domingos, J., Alobaidi, A., Kandukuri, P. L., Chaudhari, V. S., Patel, A. B., Parra, J. C., Pike, J., & Antonini, A. (2023). Impact of advanced Parkinson’s disease on caregivers: An international real-world study. *Journal of Neurology*, 270(4), 2162–2173. 10.1007/s00415-022-11546-5 36633671 PMC9835744

[cit0055] Mason, M. (2010). Sample size and saturation in PhD studies using qualitative interviews. *Forum: Qualitative Social Research*, 11(3), 1–19. 10.17169/fqs-11.3.1428

[cit0056] McDonald, C., Gordon, G., Hand, A., Walker, R. W., & Fisher, J. M. (2018). 200 years of Parkinson’s disease: What have we learnt from James Parkinson? *Age and Ageing*, 47(2), 209–214. 10.1093/ageing/afx196 29315364

[cit0057] McGinley, S., Wei, W., Zhang, L., & Zheng, Y. (2021). The state of qualitative research in hospitality: A 5-year review 2014 to 2019. *Cornell Hospitality Quarterly*, 62(1), 8–20. 10.1177/1938965520940294

[cit0058] Modestino, E. J., Reinhofer, A., Blum, K., Amenechi, C., & O’Toole, P. (2018). Hoehn and Yahr staging of Parkinson’s disease in relation to neuropsychological measures. *Frontiers in Bioscience*, 23(7), 1370–1379. 10.2741/4649 29293439

[cit0059] Montgomery, R. J. V., & Kosloski, K. (2009). Caregiving as a process of changing identity implications for caregiver support. *Journal of the American Society on Aging*, 33(1), 47–52.

[cit0060] Morrow, R., Rodriguez, A., & King, N. (2015). Colaizzi’s descriptive phenomenological method. *The Psychologist*, 28(8), 643–644.

[cit0061] Moustakas, C. (1994). *Phenomenological research methods*. SAGE Publications.

[cit0062] National Alliance for Caregiving. (2020, May). *Caregiving in the United States 2020*. AARP.

[cit0063] Navarta‐Sánchez, M. V., Ambrosio, L., Portillo, M. C., Ursúa, M. E., Senosiain, J. M., & Riverol, M. (2020). Evaluation of a psychoeducational intervention compared with education in people with Parkinson’s disease and their informal caregivers: A quasi‐experimental study. *Journal of Advanced Nursing*, 76(10), 2719–2732. 10.1111/jan.14476 32798329

[cit0064] Neubauer, B. E., Witkop, C. T., & Varpio, L. (2019). How phenomenology can help us learn from the experiences of others. *Perspectives on Medical Education*, 8(2), 90–97. 10.1007/S40037-019-0509-2 30953335 PMC6468135

[cit0065] O’Brien, B. C., Harris, I. B., Beckman, T. J., Reed, D. A., & Cook, D. A. (2014). Standards for reporting qualitative research: A synthesis of recommendations. *Academic Medicine: Journal of the Association of American Medical Colleges*, 89(9), 1245–1251. 10.1097/ACM.0000000000000388 24979285

[cit0066] Oliffe, J. L., Kelly, M. T., Gonzalez Montaner, G., & Yu Ko, W. F. (2021). Zoom interviews: Benefits and concessions. *International Journal of Qualitative Methods*, 20, 16094069211053522. 10.1177/16094069211053522

[cit0067] Pardell-Dominguez, L., Palmieri, P. A., Dominguez-Cancino, K. A., Camacho-Rodriguez, D. E., Edwards, J. E., Watson, J., & Leyva-Moral, J. M. (2021). The meaning of postpartum sexual health for women living in Spain: A phenomenological inquiry. *BMC Pregnancy and Childbirth*, 21(1). 10.1186/s12884-021-03578-y PMC7844957 33509133

[cit0068] Perepezko, K., Hinkle, J. T., Shepard, M. D., Fischer, N., Broen, M. P. G., Leentjens, A. F. G., Gallo, J. J., & Pontone, G. M. (2019). Social role functioning in Parkinson’s disease: A mixed-methods systematic review. *International Journal of Geriatric Psychiatry*, 34(8), 1128–1138. 10.1002/gps.5137 31069845 PMC6949188

[cit0069] Perry, S. E., Borders, J. C., Dakin, A. E., & Troche, M. S. (2022). Characterizing quality of life in caregivers of people with Parkinson’s disease and dysphagia. *Dysphagia*, 37(3), 523–532. 10.1007/s00455-021-10299-z 33991229

[cit0070] Phillippi, J., & Lauderdale, J. (2018). A guide to field notes for qualitative research: Context and conversation. *Qualitative Health Research*, 28(3), 381–388. 10.1177/1049732317697102 29298584

[cit0071] Prado, L., Hadley, R., & Rose, D. (2020). Taking time: A mixed methods study of Parkinson’s disease caregiver participation in activities in relation to their wellbeing. *Parkinson’s Disease*, 2020, 7370810. 10.1155/2020/7370810 PMC7171685 32351682

[cit0072] Prizer, L. P., Kluger, B. M., Sillau, S., Katz, M., Galifianakis, N. B., & Miyasaki, J. M. (2020). The presence of a caregiver is associated with patient outcomes in patients with Parkinson’s disease and atypical parkinsonisms. *Parkinsonism & Related Disorders*, 78, 61–65. 10.1016/j.parkreldis.2020.07.003 32736164

[cit0073] Roberts, R. E. (2020). Qualitative interview questions: Guidance for novice researchers. *The Qualitative Report*, 25(9), 3185–3203. 10.46743/2160-3715/2020.4640

[cit0074] Rochelle, T. L., Yeung, D. K. Y., Bond, M. H., & Li, L. M. W. (2015). Predictors of the gender gap in life expectancy across 54 nations. *Psychology, Health & Medicine*, 20(2), 129–138. 10.1080/13548506.2014.936884 25005485

[cit0075] Rodriguez, A., & Smith, J. (2018). Phenomenology as a healthcare research method. *Evidence-Based Nursing*, 21(4), 96–98. 10.1136/eb-2018-102990 30201830

[cit0076] Rose, J., & Johnson, C. W. (2020). Contextualizing reliability and validity in qualitative research: Toward more rigorous and trustworthy qualitative social science in leisure research. *Journal of Leisure Research*, 51(4), 432–451. 10.1080/00222216.2020.1722042

[cit0077] Rosqvist, K., Schrag, A., Odin, P., & The, C. C. (2022). Caregiver burden and quality of life in late stage Parkinson’s disease. *Brain Sciences*, 12(1), 111. 10.3390/brainsci12010111 35053854 PMC8773513

[cit0078] Ryan, R. M., & Deci, R. (2000). Self-determination theory and the facilitation of intrinsic motivation, social development, and well-being. *American Psychologist*, 55(1), 68. 10.1037/0003-066X.55.1.68 11392867

[cit0079] Sandelowski, M. (1995). Sample size in qualitative research. *Research in Nursing & Health*, 18(2), 179–183. 10.1002/nur.4770180211 7899572

[cit0080] Sanders, C. (2003). Application of Colaizzi’s method: Interpretation of an auditable decision trail by a novice researcher. *Contemporary Nurse*, 14(3), 292–302. 10.5172/conu.14.3.292 12868668

[cit0081] Saunders, B., Sim, J., Kingstone, T., Baker, S., Waterfield, J., Bartlam, B., Burroughs, H., & Jinks, C. (2018). Saturation in qualitative research: Exploring its conceptualization and operationalization. *Quality & Quantity*, 52(4), 1893–1907. 10.1007/s11135-017-0574-8 29937585 PMC5993836

[cit0082] Savishinsky, J. (2002). Creating the right rite of passage for retirement. *Generations*, 26(2), 80.

[cit0083] Savundranayagam, M. Y., & Montgomery, R. J. V. (2010). Impact of role discrepancies on caregiver burden among spouses. *Research on Aging*, 32(2), 175–199. 10.1177/0164027509351473

[cit0084] Schwartz, R., Zulman, D., Gray, C., Goldstein, M. K., & Trivedi, R. (2020). “It’s a disease of families”: Neurologists’ insights on how to improve communication and quality of life for families of Parkinson’s disease patients. *Chronic Illness*, 16(3), 201–211. 10.1177/1742395318799852 30208725

[cit0085] Skorvanek, M., Martinez-Martin, P., Kovacs, N., Rodriguez-Violante, M., Corvol, J.-C., Taba, P., Seppi, K., Levin, O., Schrag, A., Foltynie, T., Alvarez‐Sanchez, M., Arakaki, T., Aschermann, Z., Aviles‐Olmos, I., Benchetrit, E., Benoit, C., Bergareche‐Yarza, A., Cervantes‐Arriaga, A. … Goetz, C. G. (2017). Differences in MDS-UPDRS scores based on Hoehn and Yahr stage and disease duration. *Movement Disorders Clinical Practice*, 4(4), 536–544. 10.1002/mdc3.12476 30363418 PMC6174385

[cit0086] Smith, E. R., Perrin, P. B., Tyler, C. M., Lageman, S. K., & Villasenor, T. (2019). Parkinson’s symptoms and caregiver burden and mental health: A cross-cultural mediational model. *Behavioural Neurology*, 2019, 1–10. 10.1155/2019/1396572 PMC6913294 31871491

[cit0087] Smith, L. J., & Shaw, R. L. (2017). Learning to live with Parkinson’s disease in the family unit: An interpretative phenomenological analysis of well-being. *Medicine, Health Care and Philosophy*, 20(1), 13–21. 10.1007/s11019-016-9716-3 27364754 PMC5318469

[cit0088] Starks, H., & Brown Trinidad, S. (2007). Choose your method: A comparison of phenomenology, discourse analysis, and grounded theory. *Qualitative Health Research*, 17(10), 1372–1380. 10.1177/1049732307307031 18000076

[cit0089] Sundler, A. J., Lindberg, E., Nilsson, C., & Palmér, L. (2019). Qualitative thematic analysis based on descriptive phenomenology. *Nursing Open*, 6(3), 733–739. 10.1002/nop2.275 31367394 PMC6650661

[cit0090] Supaporn, K., Isaramalai, S., & Khaw, T. (2022). Family caregivers’ perceptions of caring for older persons in the palliative care stage at home. *Pacific Rim International Journal of Nursing Research*, 26(1), 161–174.

[cit0091] Theed, R., Eccles, F., & Simpson, J. (2017). Experiences of caring for a family member with Parkinson’s disease: A meta-synthesis. *Aging & Mental Health*, 21(10), 1007–1016. 10.1080/13607863.2016.1247414 27802771

[cit0092] Thieken, F., & van Munster, M. (2021). Deriving implications for care delivery in Parkinson’s disease by co-diagnosing caregivers as invisible patients. *Brain Sciences*, 11(12), 1629. 10.3390/brainsci11121629 34942931 PMC8699371

[cit0093] Torny, F., Videaud, H., Chatainier, P., Tarrade, C., Meissner, W. G., & Couratier, P. (2018). Factors associated with spousal burden in Parkinson’s disease. *Revista de Neurologia*, 174(10), 711–715. 10.1016/j.neurol.2018.01.372 30032927

[cit0094] Turney, F., & Kushner, B. (2018). The experience of the spouse caring for a partner with Parkinson’s disease. *Nursing Praxis in New Zealand*, 33(1), 7–16. 10.36951/NgPxNZ.2017.002

[cit0095] van Manen, M. (2017). Phenomenology in its original sense. *Qualitative Health Research*, 27(6), 810–825. 10.1177/1049732317699381 28682720

[cit0096] Vatter, S., McDonald, K. R., Stanmore, E., Clare, L., McCormick, S. A., & Leroi, I. (2018). A qualitative study of female caregiving spouses’ experiences of intimate relationships as cognition declines in Parkinson’s disease. *Age & Ageing*, 47(4), 604–610. 10.1093/ageing/afy049 29617933 PMC6014155

[cit0097] Walga, T. K. (2019). Understanding the experience and perspectives of Parkinson’s disease patients’ caregivers. *Rehabilitation Research and Practice*, 2019, 1–9. 10.1155/2019/3082325 PMC6374795 30838135

[cit0098] Watson, J. (1999). *Human science and human care: A theory of nursing*. Jones & Bartlett Learning.

[cit0099] Watson, J. (2012). *Human caring science*. Jones & Bartlett Learning.

[cit0100] White, D. R., & Palmieri, P. A. (2022). Women caring for husbands living with Parkinson’s disease: A phenomenological study protocol. *Journal of Personalized Medicine*, 12(5), 659. 10.3390/jpm12050659 35629082 PMC9146827

[cit0101] Willis, A. W., Roberts, E., Beck, J. C., Fiske, B., Ross, W., & Savica, R., et al. (2022). Incidence of Parkinson disease in North America. *NPJ Parkinson’s Disease*, 8(1), 170. 10.1038/s41531-022-00410-y PMC9755252 36522332

[cit0102] Willis, A. W., Schootman, M., Evanoff, B. A., Perlmutter, J. S., & Racette, B. A. (2011). Neurologist care in Parkinson disease: A utilization, outcomes, and survival study. *Neurology*, 77(9), 851–857. 10.1212/WNL.0b013e31822c9123 21832214 PMC3162639

[cit0103] Zucca, M., Isella, V., Lorenzo, R. D., Marra, C., Cagnin, A., Cupidi, C., Bonanni, L., Laganà, V., Rubino, E., Vanacore, N., Agosta, F., Caffarra, P., Sambati, R., Quaranta, D., Guglielmi, V., Appollonio, I. M., Logroscino, G., Filippi, M. … Rainero, I. (2021). Being the family caregiver of a patient with dementia during the coronavirus disease 2019 lockdown. *Frontiers in Aging Neuroscience*, 13. 10.3389/fnagi.2021.653533 PMC8098661 33967740

