# Supplementary material for: There is ‘no cure for caregiving’: the experience of women caring for husbands living with Parkinson’s disease
Source: Int J Qual Stud Health Well-being. 2024 Apr 24;19(1):2341989. doi: 10.1080/17482631.2024.2341989 (PMC11044767; doi:10.1080/17482631.2024.2341989)
Supplement: IJQSHW_SUPPFILE01_20230523_v1_FINAL.docx [file ZQHW_A_2341989_SM8323.docx]

**Supplemental File 1**

*Expanded Quotes by Themes and Subthemes*

**Overarching Theme: There is No Cure for Caregiving**

*“I don't know if there's anything in any study that could help the caregiver. What could you people possibly do for a caregiver? I don't know. I have my son-in-law coming in and relieving me sometimes which is the only thing that you need a break. I think God for that, I do have a break, but there is no cure for caregiver. There is no cure.”* (Rose)

| **Theme** | **Subtheme** | **Quote** |
| --- | --- | --- |
| **Caregiver Who?** |  | - *“We changed to a different neurologist, and we like this one really well, but I don't think either of them have really addressed... I don't know. The caregiver.”* (Iris) |
|  | Early years | - *"I was angry at that doctor, I thought, you've taken a vow to help people. Like, couldn't you have been kinder or more encouraging?"* and later said *"I kind of denied and didn't believe the doctor. And the doctor was saying, "No, you can't drive anymore, buddy, there's nothing we can do to help you, it's all downhill from here."* (Lilac) - *"I was in denial until I couldn't deny it anymore."* (Rose) - *"This couldn't happen to him." He was a man's man, and he had always been a coach and a teacher."* (Magnolia) - *"Fear. I think fear was the biggest. What was coming? How would I handle it? Where was my support going to come from? Would I have time for my own self? How was his life going to change? How would he handle that as well? Because neither of us knew anybody with it."* (Poppy) - *"He just told him everything, everything that's going to shut down, didn't give him any encouragement as to how he would be able to go ahead and live a fairly normal life. So, my husband was quite distraught."* (Petunia) - *"I think it's a safety mechanism in my own head, to kind of think, well, can I cope with everything alone when he leaves me? And what do I do if he gets to that fifth stage where he's on a feeding tube, and he can't speak and he can't get himself in and out of bed? And where's the adage where I can't any longer cope with it by myself?"* (Lilac) - *"I went to the library and brought home every book I could on Parkinson's, tucked it in a drawer, and read it when [my husband] wasn't around. He does not process; he does immediate head on like I do. I got myself informed and kind of ready, it took me about three years to swallow the fact that he at Parkinson's and what the future would look like. After that, I kind of griped in well. That's how we started."* (Violet) - *“I thought, "Okay, here we go." The first thing I did, I'm a type A personality, so as soon as I got home, I started researching everything I could on it, and figuring out what we could, and then trying to figure out finding a different doctor.”* (Petunia) - *"His neurologist, as we went in and they had already done all the MRIs to see if there were brain tumors and everything as they normally do it, we came in and he looked at him and he said, "Look, pal, you've got Parkinson's." We got no pamphlets. We got no written information. We got no direction as to where to look for information or research* - *“…which both of us are big researchers ... As to what does this disease mean for our life."* (Poppy) |
|  | More caring, less curing | - *"The hardest part is getting the doctors to have more time to listen to what's actually going on and not assume everything is Parkinson's."* (Poppy) - *"I'll be really honest with you, Dr. White, that guy did not even make eye contact with me. It was like I didn't exist to him, there was no instruction on what to do. I do believe that that person was a very poorly trained and I don't know, just on the very low end, like one star out of five as far as his ability to do his job."* (Lilac) - *"About him, what's coming, what needs to be done, yeah. No, the caregiver has really never come into this. As far as concentrating his practice, the caregiver doesn't really enter in unless that's an integral part of one of the problems that that person's having, is because a caregiver is in burnout, then he'll address it."* (Lily) - *"The doctors just pretty much deal with what's right in front of them. They don't go beyond there, other than to say, "You need help, go find it."* (Violet) - *"Our first neurologist was here. He is supposed to be one of the better ones, but we found that he didn't listen. And I would talk to him about things, and he would never really address the issues*." (Daisy) - *"His neurologist, as we went in and they had already done all the MRIs to see if there were brain tumors and everything as they normally do it, we came in and he looked at him and he said, "Look, pal, you've got Parkinson's." We got no pamphlets. We got no written information. We got no direction as to where to look for information or research ... Which both of us are big researchers ... As to what does this disease mean for our life."* (Poppy) - *"It would be great to compile all the data of what's available in our community for different illnesses and print up information and get it to the doctors because you're left on your own to find out. And I know it's our responsibility, but I think the doctors have a responsibility to help their patients through the journeys of their illnesses. So, I think that would be very beneficial."* (Daisy) |
|  |  |  |
|  | Living in the shadows | - *"The health providers give me lists, say, "Go find something."* (Violet) - *"I wasn't real impressed with him because when we would go in for an appointment, all of his conversation and questions were between him and my husband. And it was like, I wasn't even there."* (Carnation) - *"As a sidebar issue, maybe once or twice, but not on a regular basis. The only time I get asked that is when I go to the doctor, for me, not necessarily for him*." (Lavender) - *" It's hard because being the caregiver, that's your main focus. And you don't really think about yourself very much. You really don't because when you're watching a loved one, you push your feelings aside because you want them taken care of, and you want to make sure they have everything they need, and you don't... I don't really think about it that much, about myself that much. So, it's hard to talk about it. It's been kind of frustrating when they don't listen to you. I don't think I know more than them, but they just don't listen."* (Daisy) |
| **Taking it Day by Day** |  | - *“There’re days when I'm just exhausted. But I have learned that as I'm getting older, I can't do as much. I don't have the energy, and so I have to do what I have to do.”* (Carnation) |
|  | Golden years | - *"This was not the way I expected to spend my retirement years because we haven't been able to travel and go places a lot. Life was just all different then. And you just have no idea that that's all going to go away and there's going to be a different lifestyle."* (Carnation) - *"It's affected both our lives because we can't do the things we want to do."* (Daisy) - *"He doesn't ever want to do anything. If he had his way, he would just stay at home and do nothing. I'm not ready to give up on life yet. We used to do a lot of things. We were RVers, we did a lot of things, and he just doesn't want to do it or he's not really able to do it well. I mean I have to do it all. We're going to do it, I have to do it and I have to force the issue. That's totally different. I mean we used to be pretty adventuresome and we used to travel. My husband went to school in Germany, he's a big traveler, but he doesn't want to do anything anymore."* (Lily) - *"This isn't what we expected when I retired for our golden years. There's days when I worry about what it's going to look like down the road. I just feel like we need to enjoy what freedom we do have because in a few years, it could be worse."* (Iris) - *"I guess we both thought we'd be able to travel a little more in retirement. But because of his limitations, the only travel we've done is day trips around Colorado, not long-term trips. Probably, that's the one thing we thought we would be able to do more of, is travel, and that hasn't happened. But again, it is what it is."* (Magnolia) |
|  | Who’s gonna help me? | - *"The problem with just doing it on Zoom is they can't. They say, "Oh, you look so good. Everything's so good. You're great." They don't like live with it. And so then it's like, hard for them to understand."* (Lilac) - *"They have recommended that I joined this caregiver program, which is nationwide and what I have seen, so what I was looking for at the time was some counseling. And we are beginning to get to that where we can do some online counseling."* (Crocus) - *"I've got my brother, who's also a caregiver and he was a medical assistant as well. So, we can talk shop. We can talk. We can relate in almost every way. Between my son and daughter, we divide up some of the housekeeping stuff. I do most of the direct care and they do most of the housekeeping, helping out with meals."* (Lavender) - *"We have two daughters. One's in Oregon and one's in Denver. And the one in Denver, her and her husband, they're very good about anytime we need help, they said, just let them know and they'll come down and help us."* (Iris) - *"We do have a son here in Colorado Springs, but he's still across town and works and has kids and he does things to help when I need."* (Carnation) - *"The local Colorado Springs Parkinson's Group, we attend that on a regular basis, and we are members at large on their board. Of course, we've gathered the most strength from that." (*Petunia) - *"The support from them, that's the great thing about the support group, everybody's in the same boat and comradery really helps a lot, to know that you're not alone and everybody's facing these things."* (Lily) - *"We made fabulous friends here and they love"* my husband. They come over because *"Thursday is canasta day in my house"* and they won't let me leave him. (Rose) - *"I think the most support we get is from the Parkinson's support group."* (Daisy) - *"Last fall, we were able to get involved with" the VA. The "VA has what they call a respite program, What the veteran can and cannot do for himself, they will allow X many hours per week for someone to come into the home and help, either help the veteran himself or to help take some of the household chores and do some of those for what he worked out to be six hours a week, and we spread it out six hours every other week, because I was having someone come in and clean house for me twice a month. And they would do the grocery shopping or take him to an appointment if they had the right driver's license. And they would do some stuff around the house for me, but a lot of it was just some light stuff. And that helps."* (Crocus) |
|  | Climbing the mountain | - *"At first, that was really scary for me, which is why I eventually had to quit working outside of the home because I was afraid for him. He was unstable and there's stuff, circling. The vultures were circling, and I could recognize that, but I didn't want to leave him alone."* (Lavender) - *"I'm a very creative person, I am writing a book, which I've had to kind of put-on hold for 10 years. I feel that I've had to just let go of a lot of my own personal goals and ambitions for my own work. Which is okay, because I feel very grateful for the careers that I've had. And what I have been able to leave behind as a legacy."* (Lilac) - *"My whole life is different, but then, what's the alternative? I pray for every day, another day, another day. Please keep him the same. I don't want him getting worse."* (Rose) - *"I'm drinking more. I would have a margarita with my friend in the pool, but now it's like I just need something to take the edge off."* (Poppy) - *"You kind of don't have a choice, you have to adapt your life to it. I mean, what else can you do, except run away"* (Lily) - *“I eventually had to quit working outside of the home because I was afraid for him. He was unstable and there's stuff, circling. The vultures were circling, and I could recognize that, but I didn't want to leave him alone."* (Lavender) |
| **Not Sure What to do Next** |  | - *“How am I supposed to put my nurse hat on, take my nurse hat off? It's not one or the other, it's blending it constantly. And sometimes you have to add a little more butter or add a little more salt.”* (Lavender) |
|  | It’s my way now | - *"I do all the bills and all the phone calls, trim his fingernails, toenails, all that sort of thing. He used to be able to do just about anything around the house as far as electrical, or he could just do almost anything. And so now it's hard to see that he really can't do much at all because of the tremor. And so yeah, it's been an experience. It's just put more responsibility on me, that I feel like I have to make a lot of the decisions."* (Iris) - *“If you look around us, whatever you see or imagine, the caregiver does”* (Violet) - *"I am responsible for taking care of all his appointments and making sure he is getting there on time. And he's choosing not to go to the support meeting, the Mark Parkinson's Support meeting. It bothers him to be seeing other people even worse off than he is. And from that, what else would you want me to discuss? it does make you feel isolated. I'm responsible for everything around the house, everything around the yard. I am responsible for getting the trash out so total responsibility, he can't lift. He's very weak that way so I do."* (Pansy) - ***"****Making doctor's appointments, I do that because he's getting where he's having trouble with word retrieval and saying the right words. So, I do that, but he still takes care of the books. He still does the banking stuff. It takes him a longer time, but he still does it." (Magnolia)* - *"I'm finding I'm doing a lot of picking up from him when he changes clothes. And taking care of things at night that he doesn't take care of himself. And so that's one of the things I'm doing all the time. I'm making his meals. He can't do that. He is using a locker, trying to do more walking himself. I do all the driving."* (Pansy) - *"It was difficult at the beginning when I knew, when I got the sense that I was going to be the driver, I wasn't going to be driven in the car. That was a little hard to think about that that's the way life was going to change. So many things have changed that I just accept."* (Pansy) - *"He would fall asleep while he was driving."* (Violet) - *"It can be overwhelming. I'm doing the financial. He's no longer driving; I do all the driving."* (Poppy) - *"I do all the driving."* (Iris) - *"(I) totally manage his medications, I have had to take over driving 100 percent*." (Lily) |
|  | Nursing without a degree | - *"Because I am emotionally involved with this person [my husband], it's like a fine dance. It's a waltz. It's between two partners. You have to learn how to trust, give, take, small pressures. It's very nuanced in a relationship because there's no clear delineation between responsibilities and how you interact with my husband. How am I supposed to put my nurse hat on, take my nurse hat off? It's not one or the other, it's blending it constantly. And sometimes you have to add a little more butter or add a little more salt. Every day is a dance that you have to do."* (Lavender) - *"You listen all night long. Is he breathing? Is he breathing? I curl up to him and rock him, and that usually gets him to breathe again. So, you're not really getting good sleep because you're making sure they're still alive all night long."* (Poppy) - *"I do his medication, and I try to watch to make sure I ask him "Did you take your medication?" So, I'm kind of... I don't know the best description of it. I just am always monitoring him pretty much to see how he's doing, watching so I can tell if he's having a rough time. So, you get tired."* (Daisy) - *"I feel like I have taken the attitude of a doctor on call. And it's a 24/7 thing, because it might be the middle of the night, like he might suddenly his mask might not fit him well and have to adjust it or whatever. Because his hands don't work for Parkinson's."* (Lilac) - *“I really feel like I don't have any time on my own. I have to be aware of where he is, because he has started falling. I have to be aware, to make sure that he's using his walker, where he is going, how long he's been there, do I need to go check on him?”* (Pansy) |
|  | Emotional rollercoaster | - *"Probably the worst thing that really set me off about a month ago was he had been showering in one bathroom. I was getting cleaned up in another one. And when I went out into the kitchen, I opened up a drawer that has nice spatulas, fairly deep drawer. And there was a wet Depends diaper. And that really set me off. That was almost more than I could take. And of course, he said he didn't do it."* (Carnation) - *"Well, it's certainly been an adjustment that we did not expect to make, but yet going into a marriage, you know you grow old over time, and you know something's going to happen with one if not both of you. So, you just have to try to deal with it as it comes along. He should be able to lead a fairly good quality of life. The better his quality of life is, the better mine will be."* (Petunia) - *"I don't deserve to be spoken to or treated that way because whatever you're experiencing. So, I know you have enough self-control, so I'm happy to put myself in time out and go walk on the beach." it's not always "Okay, I see the end." because you can't see the end."* (Lavender) - *"I don't see it as a particularly positive. I don't know how to make it a positive thing for anyone."* (Crocus) - *"It's kind of difficult emotionally because you see your loved one, my husband, declining a little bit. And he's not able to do some of the things he used to do although he wants to do them. And so that's really hard on me to watch that, because I'm a fixer, I want to fix everything. And unfortunately, I can't fix it. So, the only thing that I feel like I can do is be supportive, and help him with anything that we've been told will work, like exercise. Sometimes I kind of get a little emotional, because it's real hard watching."* (Daisy) - *"Listen, I now wipe his tush when he has a bowel movement. When I say I'm the mother, I'm not kidding?"* (Rose) - *"It's hard to not be around when I leave. I always make sure he's got his phone on because if he happened to fall or something."* (Iris) - *"I really have to watch him because sometimes he puts dirty clothes in the dresser drawer and then sometimes, he'll pull out clean things and put them in the dirty clothes or he'll try to put on two pair of sleep pants, that kind of thing. It's like the brain is almost frozen. And he just can't process the everyday activities. It's living with a little child that you are totally are responsible for what goes on."* (Carnation) - *"Are you kidding me?" So really you have no life but what you make of your own, and the socialization with the friends that we have, they have been beautiful, beautiful people to have met. Very fortunate in that. So daily life is what am I making for dinner? Is he dressed? He can get the shirt over his head, but he can't pull it down on the back. So, you're helping him dress. Reminding him, "Take your inhaler, take your Flonase. Did you brush your teeth before you came to bed?"* (Poppy) |
| **Just Too Much** |  | - *"Probably the worst thing that really set me off about a month ago was he had been showering in one bathroom. I was getting cleaned up in another one. And when I went out into the kitchen, I opened up a drawer that has nice spatulas, fairly deep drawer. And there was a wet Depends diaper. And that really set me off. That was almost more than I could take. And of course, he said he didn't do it."* (Carnation) |
|  | Physical and mental overload | - *"When they told us there would be some cognitive decline, we did not realize the amount of cognitive decline."* (Poppy) - *"He wakes up and he may be confused or start just talking about something that doesn't make sense. And so, that's changed life a lot because we used to go a lot, do a lot of things and can't do that anymore. That's just part of where we are in with this illness."* (Carnation) - *"Losing some of the sharp edge, whether that's just aging in general, or some beginning of some dementia, I'm not in a place where I can diagnose that, but we're noticing differences."* (Crocus) - *"Now he can hardly speak to be understood."* (Lilac) - *"The other hardest thing right now, is just this freezing, this sort of skittering steps that he takes."* (Lilac) - *"He started shuffling his feet, and I'm always reminding him "Come on, big steps, not these baby steps, because that's not good." And he goes "I'm working on it, I'm working on it*." (Daisy) - *"I can touch him and make him aware of another part of his body, it kind of wakes up the brain, I think, and he'll stop, and then the next step he takes is a good step. But if I'm not close enough to touch him, I try and say, "(honey), stop." And if he looks at me, and I say stop again, again, it's like he's activating that brain again to rethink what his legs are doing. I don't know if that makes sense."* (Magnolia) - *“His tremor, it's worse in the mornings. He has trouble getting his coffee. And so I guess that I think that would be the most challenging.”* (Iris) |
|  | I’m just so angry | - *"So, my dad is saying, "Get up and let's play dice or play cards," so we can interact. He's only here until tomorrow. But everything revolves around his needs. If you have to go to the doctor, oh, the minute you're walking out the door, "I should try to go to the bathroom." (Poppy)* - *"Well sometimes I just have to bite my lip and turn the cheek and do something else. Walk away from it because if he gets upset or angry about something, you can't push on it. It's not like you can talk it out."* (Carnation) - *"So, I struggle with my own, like, reactions of anger, like, how could you not hear the understand that or I just said that to you, I have to repeat myself a lot." (Lilac)* - *"I don't see it as a particularly positive. I don't know how to make it a positive thing for anyone."* (Crocus) - *"I'm noticing when I go out, he wants to know every place I've been. And sometimes I don't want to tell him, or I'm just ordinary things I was doing, errands. Just having friends, it's a struggle to even have friends over anymore. That's hard for me because I like to serve, I like to have dinners and enjoy company with people, and he's not so much anymore. And he is on a diet, so eating out isn't what he can do that much. And that's okay with me. I like cooking, so I don't mind eating at home. But probably the hardest part is not wanting to be with friends."* (Pansy) |
|  | Managing the craziness | - *"On bad days, I'm a bitch. I'm angry, and I'm resentful, and it's not his fault. Then I become guilty. Then at night, I'll start to cry and apologize because it's not his fault. He took care of me; it's now my turn. I know that intellectually."* (Rose) - *"I would say that it builds up to a certain point. I cry and take a long hot bath, and then we go on." I also "get a massage every other week."* (Crocus) - *"Well, like I said, it's very faith-based. If I need help, I can ask for blessings."* (Violet) - *"How I deal with the stress? Well, I pray a lot. And if I'm too stressed, I call a friend, talk to them about it. I do crafts. And so sometimes I can just get lost in that and get busy doing crafts. And so not thinking about anything else."* (Iris) - *"I pray. I just give it to the Lord, and I take it off my shoulders. That's it."* (Magnolia) - *"So, we're going out to a restaurant with everyone tonight, and then they're coming to our house for dessert, and we'll play a game or something. We try to do that. Every Thursday, we try to set it up with some friends, we play a specific game called Mexican Train. Just anything like that that will settle things down for a little while."* (Petunia) |
| **Caring is Your Souls Growth** |  | - *“One way that I looked at this is, and is outside of the kind of normal medical thing, but it's more of a spiritual thing that has helped me to say, okay what, if on a spiritual level, you signed up as a contract for your life to get some, that this is part of your soul's growth, that by helping this other person, by sacrificing and letting go of the things that you have to no longer be able to do with him together, or even dreams that I might have had for us as a couple that we can't do anymore.”* (Lilac) |
|  | Please God help me | - *"My faith being a Christian is the biggest thing that gives me inner strength* (Iris) - *"As a spirit, I believe my purpose is to be of service and that I am intelligent and can figure out. I can find a solution to all things. And my mom was a great example of it. She was just a fantastic person. She was very spiritual."* (Lavender) - *"I have God on my side, so I have family and friends who include me and their prayers."* (Carnation) - *"I pray. I just give it to the Lord, and I take it off my shoulders."* (Magnolia) - *"I am a Christian, and I have promised when I married him to love him, no matter what until we're both gone and or I'm gone right or he's gone. And I am dedicated to that. Knowing that God is with me and helping me. I don't know if someone didn't have God in their life, how they would handle it."* (Pansy) |
|  | Finding my inner strength | - *"I am really good at helping other people when it's not me. I guess the only strength that I can really pull from is the fact I understand what's happening because I've been there before."* (Lily) - *"I'm gentle but strong when I have to be, but I'm stronger than a bull. There are times when my mind goes to maybe I shouldn't have married him. When it boils down to it, I say to myself, "Would you rather have had somebody else?" No. He's, my husband. I love him."* (Rose) - *"This is just something I need to do."* (Crocus) - *"I think my faith being a Christian is the biggest thing that gives me inner strength. And friends. Just a lot of support from friends. It's part of life, and that he'd be there for me, and I want to be there for him. And I guess it's just a commitment and a strong faith."* (Iris) - *"I'm able to focus on something else other than his problems. And I think just being active, and keeping busy, and keeping my mind busy, and doing other things helps me."* (Magnolia) - *"Having to care for him has made me a much better person than I would have been. I'm kinder; I'm more compassionate; I'm more tolerant; I'm more patient; I'm more flexible. I'm less perfectionist and demanding."* (Lilac) - *"I want to continue sharing all the life experiences we can together and make sure he realizes he's still worthy. So, that's what drives me, I love him."* (Poppy) - *"A Bit of humor: He says, "Just call me Abby, Abby Normal. So, he used his sense of humor to get through things. And some days, I'll just look at him, and I'll go, "This is truly an Abby day." And he goes, "Yes, it is." That's the way I am." I think I'm still the same person. Little older, but I still have friends, I still make connections."* (Daisy) |
|  | How can I help you? | - *"One of the things that I always encourage people in our support groups to make sure they did is carry a notebook to every appointment. Put the date, the name of the doctor you're seeing, what you're there for, and your questions."* (Poppy) - *"Look for all the info that you can find as far as the disease is. Look for all the support that you can find."* (Carnation) - *"I would encourage anyone having the experience of a husband or wife with Parkinson's to learn as much as you can about it, and the causes and what do they called it? Experiences that they'll go through so that you're not unaware of that and attend as many meetings as you can in your area to have support from the group Parkinson's Group."* (Pansy) - *"I would say when they first learn that they're going to be involved with caregiving, I think if they get involved with some kind of a group that has the knowledge and resources to support them in whatever they're going to need, would be an important factor for them to consider. And just talking to other people who are in the know about what Parkinson's is going to look like as their person progresses, and just being able to know, "Okay, this might happen. This might happen."* (Magnolia) - *"There are more caregivers out there, which is, I suppose, partly in response to the aging general population and the fact that we have much greater need for more caregivers, that people are willing to do that, people who hadn't considered that before, that they're considering it as a career."* (Crocus) |
